# Araticum (*Annona crassiflora*) seed extract as a chemopreventive agent against prostate cancer: activation of extrinsic and intrinsic apoptotic pathways in a preclinical model

**DOI:** 10.1007/s10735-026-10859-3

**Published:** 2026-06-13

**Authors:** Iara Lopes Lemos, Maria Josiane Macedo, Felipe Rabelo Santos, Bianca Barbosa Rezende, Fabio Montico, Valeria Helena Alves Cagnon, Mario Roberto Marostica Junior

**Affiliations:** 1 Faculty of Food Engineering (FEA), Department of Food Science and Nutrition (DECAN), University of Campinas (UNICAMP), Campinas, São Paulo 13083-862 Brazil; 2 Department of Structural and Functional Biology, Institute of Biology, University of Campinas (UNICAMP), Campinas, São Paulo 13083‐862 Brazil

**Keywords:** Natural products, Bioactive compounds, Cancer, TRAMP mice, Cell death

## Abstract

Prostate cancer (PCa) is among the most frequently diagnosed malignancies in aging men and remains a major public health concern, highlighting the need for effective and safe preventive strategies. *Annona crassiflora* Mart. seed extract (ASE) was evaluated using the transgenic adenocarcinoma of the mouse prostate (TRAMP) model. Animals were divided into control and ASE-treated groups evaluated from 8–12 to 12–16 weeks of age. Control groups received the vehicle (water containing 10% DMSO), whereas ASE-treated groups received 100 mg/kg body weight of ASE. The preservation of hepatic histoarchitecture and normal body weight gain demonstrated that ASE was well tolerated and did not show hepatotoxic effects. ASE treatment increased the frequency of healthy prostatic epithelium in early prostate lesions (predominantly low-grade prostatic intraepithelial neoplasia (LGPIN)) and late prostate lesions (high-grade prostatic intraepithelial neoplasia (HGPIN) and well-differentiated adenocarcinoma (WDAC)) of PCa. Furthermore, ASE reduced HGPIN incidence in the ASE 12–16 group, showing a lower incidence of WDAC. In addition, ASE reduced androgen receptor (AR) protein levels as well as cytoplasmic and nuclear AR localization in late prostate lesions (HGPIN and WDAC) of PCa. ASE also induced apoptosis through multiple pathways, including upregulation of BID, BAD, and BAX, activation of caspase-8, caspase-9, and caspase-3, and downregulation of BCL-2, BCL-xL, and MCL-1. Thus, ASE promotes a pro-apoptotic environment unfavorable to tumor survival. Taken together, these results reinforce the potential of ASE to modulate prostatic homeostasis, indicating a promising strategy with chemopreventive effects and potential as an adjuvant therapy against PCa.

**Graphical Abstract:**

**Figure:**
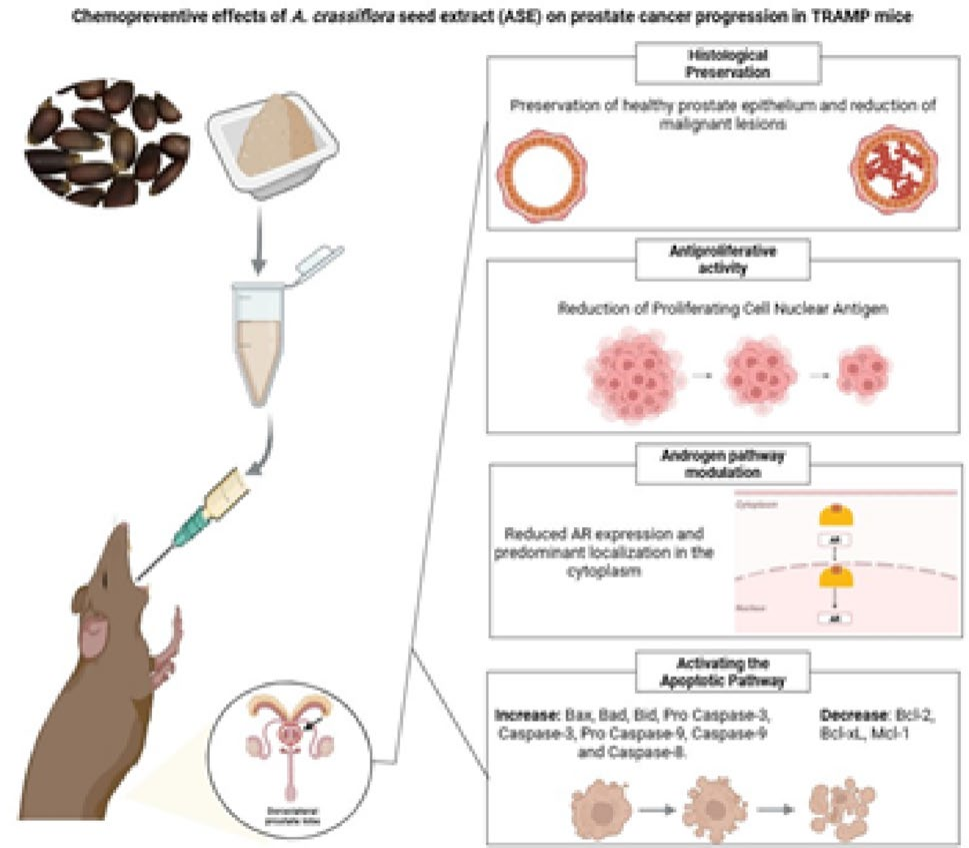

**Supplementary Information:**

The online version contains supplementary material available at 10.1007/s10735-026-10859-3.

## Introduction

Among men, prostate cancer (PCa) is as the second most frequently diagnosed malignancy and primarily affects individuals over 60 years of age (Bergengren et al. 2023). Projections indicate that 313,780 cases of PCa will be diagnosed in 2025 and that approximately 3.5 million people will be living with the disease in the United States; in addition, this number is expected to rise to 4.2 million by 2035 (Wagle et al. 2025). PCa develops from mutations in healthy cells, transforming them into cancerous cells and initially leading to the development of low-grade prostatic intraepithelial neoplasia (LGPIN), followed by high-grade prostatic intraepithelial neoplasia (HGPIN), adenocarcinoma, and, in some cases, metastasis (Saranyutanon et al. 2020; Kulac et al. 2024).

PCa development and maintenance have been associated with testosterone action, as well as its more potent form, dihydrotestosterone (DHT), which binds to the androgen receptor (AR) and activates its signaling pathways. This activation can influence cell proliferation and inhibit apoptotic mechanisms (Basak et al. 2022). Apoptosis is a form of programmed cell death that plays an important role in cancer prevention and therapy. Thus, when the apoptotic process is inhibited, tumor progression is favored due to the increased survival of cancer cells (Gupta et al. 2023). Life expectancy has increased significantly in recent decades; therefore, PCa incidence in the male population is also expected to rise, making it essential to develop new strategies for the prevention or treatment of the disease (Schatten 2018). Animal models and novel compound have proven to be effective preclinical tools for studying chemopreventive effects (Kido et al. 2019).

Nowadays, natural products and plant-derived bioactive compounds have attracted considerable attention as promising agents for cancer prevention and treatment due to their multitarget biological activities and relatively low toxicity profiles. Several phytochemicals, including polyphenols, flavonoids, terpenoids, and alkaloids, have demonstrated antioxidant, anti-inflammatory, antiproliferative, and pro-apoptotic effects in different experimental cancer models (Nelson et al. 2022; Awad et al. 2023; Bouabdallah et al. 2024). Natural compounds such as safranal, a major constituent of saffron (Crocus sativus), have been shown to induce apoptosis through redox imbalance, disruption of cellular energy homeostasis, protein destabilization, and DNA damage in tumor cells (Nelson et al. 2022). Likewise, saffron-derived compounds, including crocin, crocetin, and safranal, significantly inhibited tumor growth in prostate, cervical, liver, and lung cancer models (Awad et al. 2023). In addition, medicinal plants traditionally used in folk medicine, such as Tribulus terrestris, have emerged as important sources of bioactive compounds with anticancer, antioxidant, anti-inflammatory, and immunomodulatory activities, mainly attributed to their high content of steroidal saponins, flavonoids, tannins, and terpenoids (Bouabdallah et al. 2024). Polyphenolic compounds have also demonstrated selective anticancer effects by acting as pro-oxidants in tumor cells while exerting cytoprotective antioxidant effects in normal tissues. For example, Hibiscus sabdariffa extract enhanced the selective toxicity of cisplatin against non-small cell lung cancer cells while simultaneously protecting hepatic tissue from chemotherapy-induced toxicity, effects associated with phenolic compounds such as quercetin, rutin, chlorogenic acid, gallic acid, and caffeic acid (Hamza et al. 2023). Collectively, these findings reinforce the growing interest in natural products as promising chemopreventive and adjuvant therapeutic agents capable of modulating oxidative stress, apoptosis, and tumor progression.

Accordingly, the Transgenic Adenocarcinoma of the Mouse Prostate (TRAMP) model, which presents characteristics similar to those of human PCa, represents an interesting model for evaluating PCa progression (Kido et al. 2019). The main challenge of chemoprevention studies in PCa is to effectively delay tumor progression while avoiding adverse effects (Kido et al. 2019). In consequence, bioactive compounds present in plant species have been recognized as natural agents with chemopreventive potential against cancer (Chhabra et al., 2018). These compounds can act by preventing cellular mutations, inhibiting the uncontrolled growth of already mutated cells, and preventing the progression of premalignant lesions to malignant lesions (Chhabra et al., 2018). Importantly, in vivo models provide critical information that cannot be fully reproduced in cell culture systems, including tumor microenvironment interactions, lesion progression dynamics, tissue architecture alterations, systemic toxicity, and pharmacological responses during prostate carcinogenesis (Ittmann et al. 2013; Kido et al. 2019; Sailer et al. 2023).

Brazil has one of the richest plant biomes in the world; however, this valuable ecosystem remains underexplored. Native Brazilian species are rich sources of bioactive compounds, although the direct consumption of native Brazilian fruits and their derived products remains limited (Santos et al., 2023). Polyphenols are secondary metabolites widely distributed in fruits, spices, nuts, and other plant-derived foods and are recognized for their antioxidant, anti-inflammatory, and anticancer properties, demonstrating considerable potential for anticancer drug development (Ahmad et al. 2022).

Araticum (*Annona crassiflora* Mart.) is a native Brazilian fruit predominantly found in the Cerrado biome that remains underexplored despite its promising phytochemical composition. *A. crassiflora* can be consumed fresh or incorporated into food products (Costa et al. 2025). Previous studies have demonstrated promising effects of this species against liver and cervical cancer models (Justino et al. 2021; Rosa et al. 2021). The peel and seed are among the least explored fractions of the fruit and are commonly discarded after consumption. Nevertheless, these fractions are rich in phenolic compounds and acetogenins and exhibit high antioxidant capacity, which has stimulated interest in investigating the potential effects of these fractions against PCa (Arruda et al. 2018). To date, only two studies have investigated the effects of *A. crassiflora* in PCa, one of which was conducted by our research group (Prado et al. 2020; Lemos et al. 2025). In PCa cell lines, both peel and seed extracts reduced cell viability and induced apoptosis in LNCaP cells, with the seed extract showing the most pronounced effects (Lemos et al. 2025).

Despite these promising findings, important gaps remain regarding the chemopreventive potential of *A. crassiflora* seed extract, particularly in vivo*,* especially in prostate given its complexity. Therefore, the objective of this study was to evaluate the effects of *A. crassiflora* seed extract (ASE) on delaying tumor progression in TRAMP mice and to investigate its ability to modulate apoptotic pathways during the early prostate lesions (predominantly LGPIN) and late prostate lesions (including HGPIN and WDAC) of PCa.

## Materials and methods

### Characterization of araticum seed extract (ASE)

Ripe fruits of *A. crassiflora* were cleaned and manually peeled, and the seeds were freeze-dried. The extraction protocol was previously described by Lemos et al. (2025). Briefly, the freeze-dried and ground seeds were homogenized with a hydroalcoholic solution in a thermostatic shaking bath (Lemos et al. 2025). Detailed characterization of the phenolic compounds present in ASE and evaluation of its in vitro antioxidant activity were previously performed by the same research group. The main compounds present in ASE were identified and quantified using a mass spectrometry method based on multiple reaction monitoring (LC–MS/MS–MRM) (Lemos et al. 2025). Total phenolic compounds were also quantified using the Folin–Ciocalteu method. Antioxidant capacity was evaluated using three different assays: Oxygen Radical Absorbance Capacity (ORAC), Ferric Reducing Antioxidant Power (FRAP), 2,2′-azinobis (3-ethylbenzothiazoline-6-sulfonic acid) (ABTS). Importantly, the extract used in the present study corresponds to the same extract previously used by Lemos et al. (2025), thus ensuring methodological standardization between studies.

### Animals and experimental procedures

Sixty male TRAMP mice (C57BL/6-Tg (TRAMP)8247Ng/J × FVB/NJ F1/J) were obtained from the Multidisciplinary Center for Biological Research at the State University of Campinas (CEMIB/UNICAMP). Animals were randomly allocated into four experimental groups (n = 15/group): Control 8–12, ASE 8–12, Control 12–16, and ASE 12–16, to evaluate the ASE effects during different stages of prostate lesion progression in the TRAMP model. The chosen age intervals were based on the well-established age-dependent progression of prostate carcinogenesis in TRAMP mice, in which PIN lesions predominate between 8–12 weeks, whereas more advanced lesions, including HGPIN and adenocarcinoma, become increasingly frequent between 12–16 weeks of age (Greenberg et al. 1995; Gingrich and Greenberg 1996; Kaplan‐Lefko et al. 2003; Berman-booty et al. 2012; Kido et al. 2019).

**Fig. 1:**
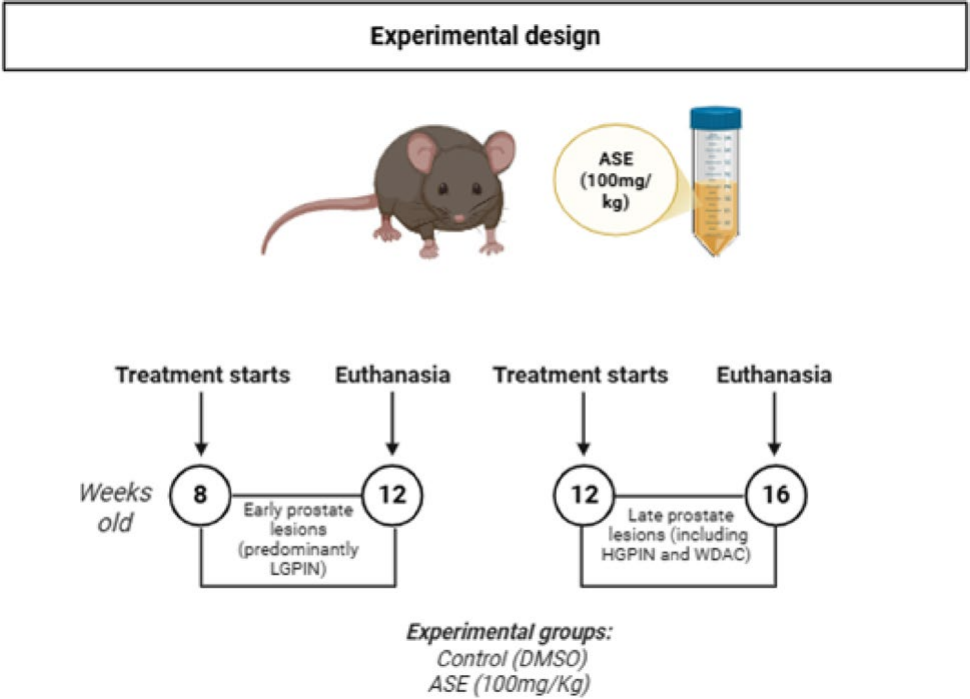
Experimental design of the TRAMP mouse study and experimental groups. Schematic illustration created using BioRender.com

The Control 8–12 and ASE 8–12 groups began treatment at 8 weeks of age and were euthanized at 12 weeks of age. The Control 12–16 and ASE 12–16 groups began treatment at 12 weeks of age and were euthanized at 16 weeks of age. ASE-treated animals received ASE at a dose of 100 mg/kg body weight diluted in 10% DMSO by oral gavage, five times per week for four weeks. Control animals received only the vehicle solution (10% DMSO diluted in water) under the same administration protocol (Fig. 1). Due to the low aqueous solubility of ASE, DMSO was used to ensure adequate solubilization and homogeneous administration throughout the experimental period. Similar concentrations of DMSO have been previously employed in in vivo studies involving plant-derived extracts and hydrophobic compounds, without evidence of significant systemic toxicity (Colucci et al. 2008; Nasir et al. 2020; Emiru et al. 2021). During the experimental period, the animals were monitored daily for signs of distress or toxicity, including behavioral alterations, mortality, and body weight changes. In addition, liver analyses were performed to investigate potential treatment-related systemic toxicity. These data are presented in the Supplementary Material.

The dosing criteria were based on previous preclinical studies involving species of the Annonaceae family, particularly *Annona muricata*, which presents phytochemical characteristics similar to ASE (Yang et al. 2015; Ebenyi et al. 2024). In addition, the chosen ASE dose (100 mg/kg body weight) was defined based on preliminary pilot experiments conducted by our research group using different concentrations (50 and 100 mg/kg body weight) in the TRAMP model. Based on the initial efficacy and safety observations obtained in these experiments, the 100 mg/kg dose was chosen for further mechanistic investigation of PCa progression. During the experimental period, animals were monitored daily for clinical signs of toxicity or distress, including behavioral alterations, mortality, and body weight changes. In addition, histopathological analyses of the liver were performed to evaluate potential systemic toxicity associated with ASE or vehicle administration, as described in the Supplementary Material.

After the treatment period, animals were weighed using a Denver P-214 analytical balance (Denver Instrument Company, Arvada, CO, USA), anesthetized with 2% xylazine hydrochloride (5 mg/kg, i.m.; König, São Paulo, Brazil) and 10% ketamine hydrochloride (60 mg/kg, i.m.; Fort Dodge, IA, USA), and euthanized by cardiac puncture under deep anesthesia. The dorsolateral prostate lobes were collected for subsequent analyses (CEUA no. 6174–1/2023).

### Histopathological analysis 

Samples from dorsolateral lobe of the prostate of five animals per group were fixed in Bouin’s solution, dehydrated in ethanol, cleared in xylene, and embedded in Paraplast, following previously established protocols (Rossetto et al. 2023; Montico et al. 2023; Santos et al. 2024). Sections (5 µm) were obtained using a Hyrax M60 microtome (Zeiss), mounted with 3–4 sections per slide, stained with hematoxylin and eosin, and imaged at 40 × magnification (Nikon Eclipse E-400, Tokyo, Japan).

Histopathological evaluation was performed using ten randomly selected microscopic fields per animal. Each prostatic region was divided into four quadrants using NIS-Elements (Nikon) imaging software. In each quadrant, epithelial alterations were classified according to a predefined grading scale as follows: (1) healthy epithelium, (2) low-grade prostatic intraepithelial neoplasia (LGPIN), (3) high-grade prostatic intraepithelial neoplasia (HGPIN), and (4) well-differentiated adenocarcinoma (WDAC), based on Gingrich et al. (1999) and Berman-Booty et al. (2012). Healthy epithelium was characterized by a monolayer of cuboidal to columnar cells with basally located round nuclei; LGPIN by epithelial proliferation with an increased nucleus-to-cytoplasm ratio, elongated nuclei, and condensed chromatin; HGPIN by epithelial stratification with papillary or cribriform projections without stromal invasion; and WDAC by acinar and tubular structures with nuclear and cytoplasmic atypia, high mitotic index, and stromal invasion (Gingrich et al. 1999; Berman-booty et al. 2012; Kido et al. 2016; Nogueira Pangrazi et al. 2018). The relative frequency of each histopathological pattern was calculated as the proportion of each classification relative to the total number of evaluated foci within each experimental group.

**Fig. 2:**
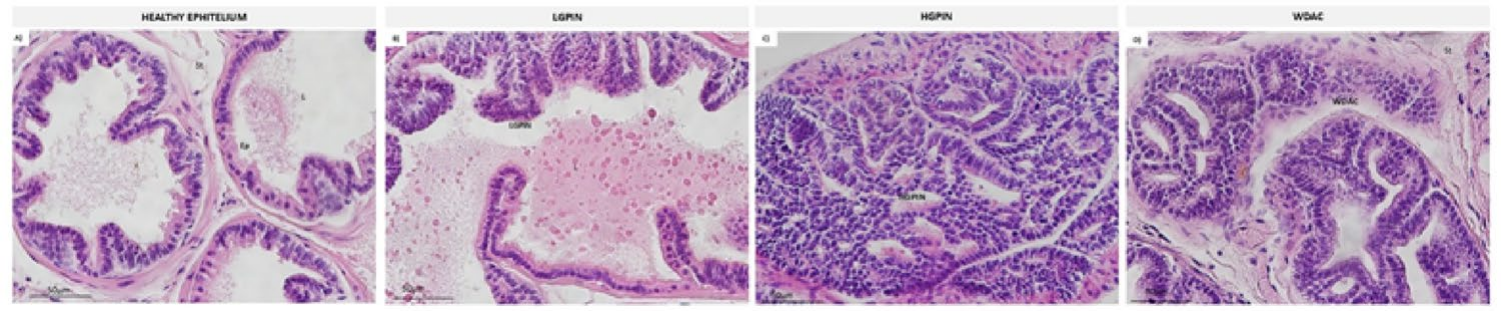
Representative photomicrographs of cancer progression in the dorsolateral lobe of the TRAMP mice. **Abbreviations:** EP: Healthy Epithelium, HGPIN: high-grade prostatic intraepithelial neoplasia, L: lumen, LGPIN: low-grade prostatic intraepithelial neoplasia, St: stroma. WDAC: Well-differentiated adenocarcinoma

Representative morphological features of each lesion type are shown in Fig. 2.

## Immunohistochemistry

Prostate samples from the dorsolateral lobe of five animals per group were processed for immunohistochemical analysis of caspase-3, BID, Bcl-xL, PCNA, and AR, following the protocols described by Montico et al. (2023) and Rossetto et al. (2023). Immunoreactivity for Bcl-xL, BID, and caspase-3 was semiquantitatively scored (0–3) based on staining intensity and extent in ten fields at 400× magnification, as described by Tuxhorn et al. (2002). The final reactivity score was obtained by multiplying both indices and classified as follows: 0 = absent; 1–3 = weak; 4–6 = moderate; and 7–9 = strong. Results were expressed as the percentage of animals in each group exhibiting each reactivity pattern (Tuxhorn et al. 2002; Montico et al. 2023; Rezende et al. 2025). Negative controls and antibody labeling details are provided in the Supplementary Material. Detailed information on the antibodies used, including dilution and supplier, is described in Table 1.

**Table 1:** Primary and secondary antibodies used for Immunohistochemistry analysis, including dilution and reference

Antibody	Classification	Range dilution	Reference
Anti-mouse	Secondary	1:100—1:200	W4021, Promega Corporation
Anti-rabbit	Secondary	1:200	W4018, Promega Corporation
AR	Primary	1:100	ab74272, Abcam
BID	Primary	1:200	Ab32060, Abcam
BCL-XL	Primary	1:300	2764S, Cell Signalling
Caspase-3	Primary	1:100	Sc56053, Santa Cruz Biotechnology
PCNA	Primary	1:500	ab29, Abcam

### Western blotting analysis

Samples from the dorsolateral prostate lobe of TRAMP mice (n = 5 per group) were collected and processed for protein extraction as previously described by Lemos et al. (2025), Rossetto et al. (2023), and Santos et al. (2024). Pixel densitometry analysis was performed using Uni-Scan-It 6.1 software, with β-actin used as an endogenous control (Rossetto et al. 2023; Santos et al. 2024; Lemos et al. 2025). Detailed information on the antibodies used, including dilution and supplier, is described in Table 2.

**Table 2:** Primary and secondary antibodies used in Western blotting, including antibody classification, dilution range, and reference

Antibody	Classification	Range dilution	Reference
Anti-mouse	Secondary	1:2000—1:5000	W4021, Promega Corporation
Anti-rabbit	Secondary	1:2000—1:5000	W4018, Promega Corporation
AR	Primary	1:500	ab74272, Abcam
BAX	Primary	1:250	ab32503, Abcam
BCL-2	Primary	1:250	CS3869, Cell Signalling
BID	Primary	1:250	Ab32060, Abcam
MCL-1	Primary	1:500	5453S, Cell Signalling
BAD	Primary	1:250	9292S, Cell Signalling
BCL-XL	Primary	1:1000	2764S, Cell Signalling
Caspase-3	Primary	1:500	Sc-56053, Santa Cruz Biotechnology
Caspase-8	Primary	1:1000	4790S, Cell Signalling
Caspase-9	Primary	1:500	Sc-56076, Santa Cruz Biotechnology
PCNA	Primary	1:500	ab29, Abcam
β-actin	Primary	1:500	Sc 81,178, Santa Cruz Biotechnology

### Statistical analysis

One-way ANOVA followed by Tukey’s post hoc test was used for all statistical analyses performed using GraphPad Prism software (version 8.01). Data were previously confirmed as parametric using the Shapiro–Wilk test, and statistical significance was set at p < 0.05 (Montgomery 1991; Zar 1999).

## Results

### Evaluation of treatment tolerability and safety

To assess tolerability and potential toxic effects associated with ASE treatment and its dilution vehicle (water containing 10% DMSO), objective parameters of general health were monitored throughout the experimental period. Administration of ASE (100 mg/kg body weight) or its vehicle, 5 days per week, did not result in changes in body weight, and the animals exhibited progressive and comparable weight gain among groups, regardless of age. In addition, liver histopathological analysis did not reveal structural alterations indicative of hepatotoxicity. Detailed data regarding body weight monitoring and hepatic histological evaluation are presented in the Supplementary Material (Figures S1, S2 and S3).

### ASE exerted chemopreventive effects and attenuated malignant lesions and cell proliferation

**Fig. 3:**
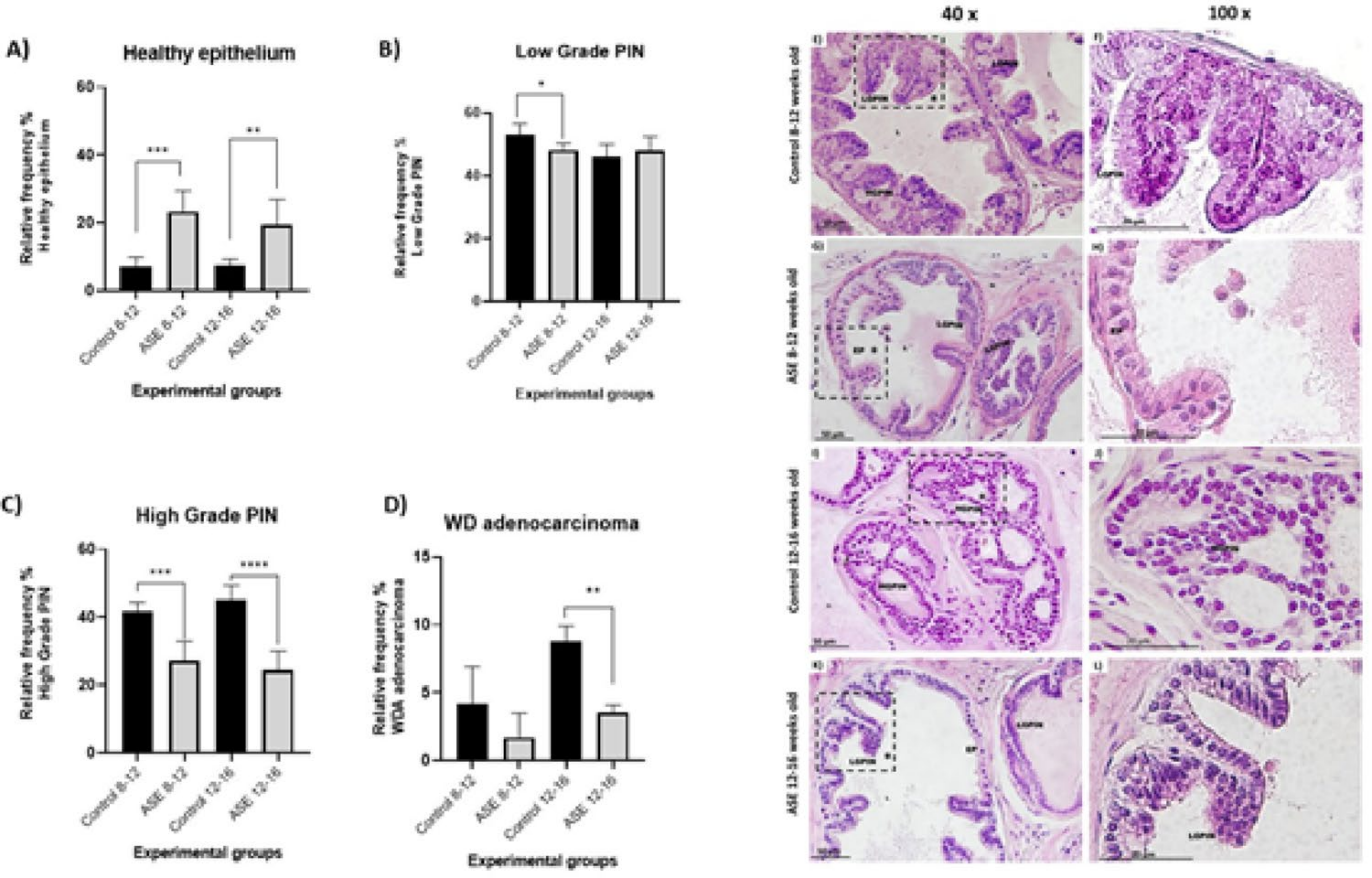
**A**–**D**: Relative frequency (%) of healthy epithelium (**A**), LGPIN (**B**), HGPIN (**C**), and WD adenocarcinoma (**D**). E–L: Representative photomicrographs of the main histopathological findings observed in the Control 8–12 (**E**–**F**), ASE 8–12 (**G**–**H**), Control 12–16 (**I**–**J**), and ASE 12–16 (**K**–**L**) groups. Left panels (**E**, **G**, **I**, and **K**) show images at 40 × magnification, whereas right panels (**F**, **H**, **J**, and **L**) show higher magnification (100 ×) images of the regions indicated by dashed rectangles in the corresponding left panels. Sections were stained with hematoxylin and eosin (H&E). Data are presented as mean ± SD (n = 5 per group). Statistical significance: *p < 0.05, **p < 0.01, ***p < 0.001, ****p < 0.0001. Abbreviations: ASE, Araticum seed extract; HGPIN, high-grade prostatic intraepithelial neoplasia; L, lumen; LGPIN, low-grade prostatic intraepithelial neoplasia; St, stroma

ASE treatment increased the frequency of healthy epithelium in animals across all experimental groups (Fig. 3A). In the ASE 8–12 group, a reduction in both LGPIN and HGPIN lesions was observed (Fig. 3B and C). Regarding WDAC, no statistical difference was detected between the Control 8–12 and ASE 8–12 groups. In older animals (12–16 weeks of age), no changes were observed in LGPIN lesions (Fig. 3B). However, ASE treatment reduced the incidence of HGPIN lesions (Fig. 3C). In addition, the ASE 12–16 group showed a lower incidence of WDAC (3.53% ± 0.55) compared with the respective control group (8.7% ± 1.14) (Fig. 3D).

**Fig. 4:**
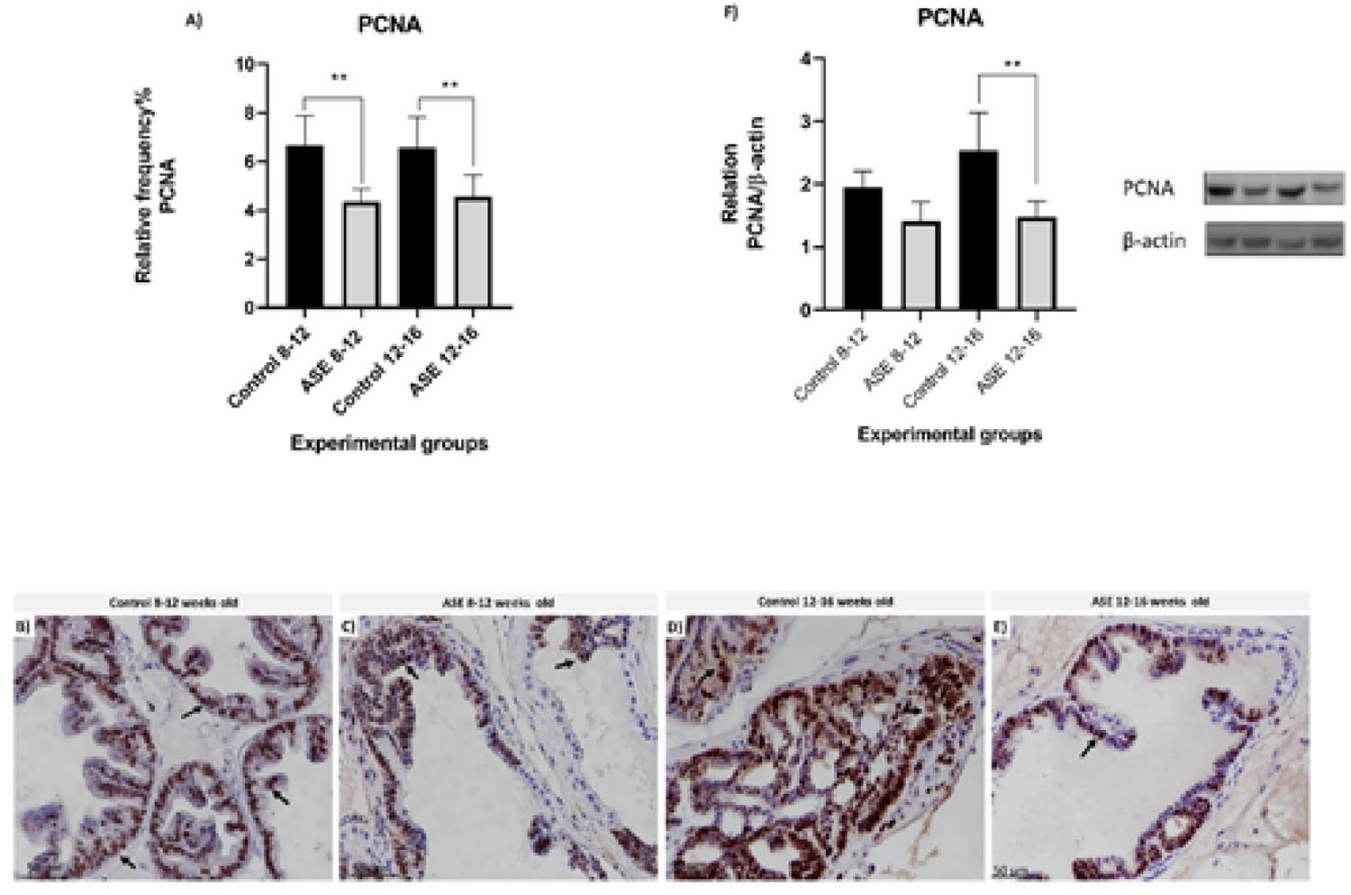
Immunohistochemical analysis and protein expression of PCNA levels in the dorsolateral lobe of the TRAMP mouse prostate. **A** Relative frequency (%) of PCNA-positive cells in the 8–12- and 12–16-week-old groups. **B–E** Representative photomicrographs of PCNA immunostaining in the Control 8–12 **B**, ASE 8–12 (**C**), Control 12–16 (**D**), and ASE 12–16 (**E**) groups. Images were obtained at 40 × magnification and counterstained with hematoxylin. Arrows indicate positive PCNA immunostaining. **)** Western blot analysis of PCNA protein expression in the 8–12- and 12–16-week-old groups, with representative bands of protein blots shown on the right. Data are presented as mean ± SD. Statistical significance was considered between the Control and ASE groups within the same age period (*p < 0.05; **p < 0.01)

Regarding cell proliferation, reduced PCNA immunolocalization was observed in the ASE-treated groups at all evaluated ages (Fig. 4A, B, C, D and E). However, a decrease in PCNA protein levels, as determined by Western blot analysis, was observed only in animals aged 12–16 weeks (Fig. 4F).

### ASE reduces AR expression and influences its cytoplasmic retention in early prostate lesions (predominantly LGPIN)

**Fig. 5:**
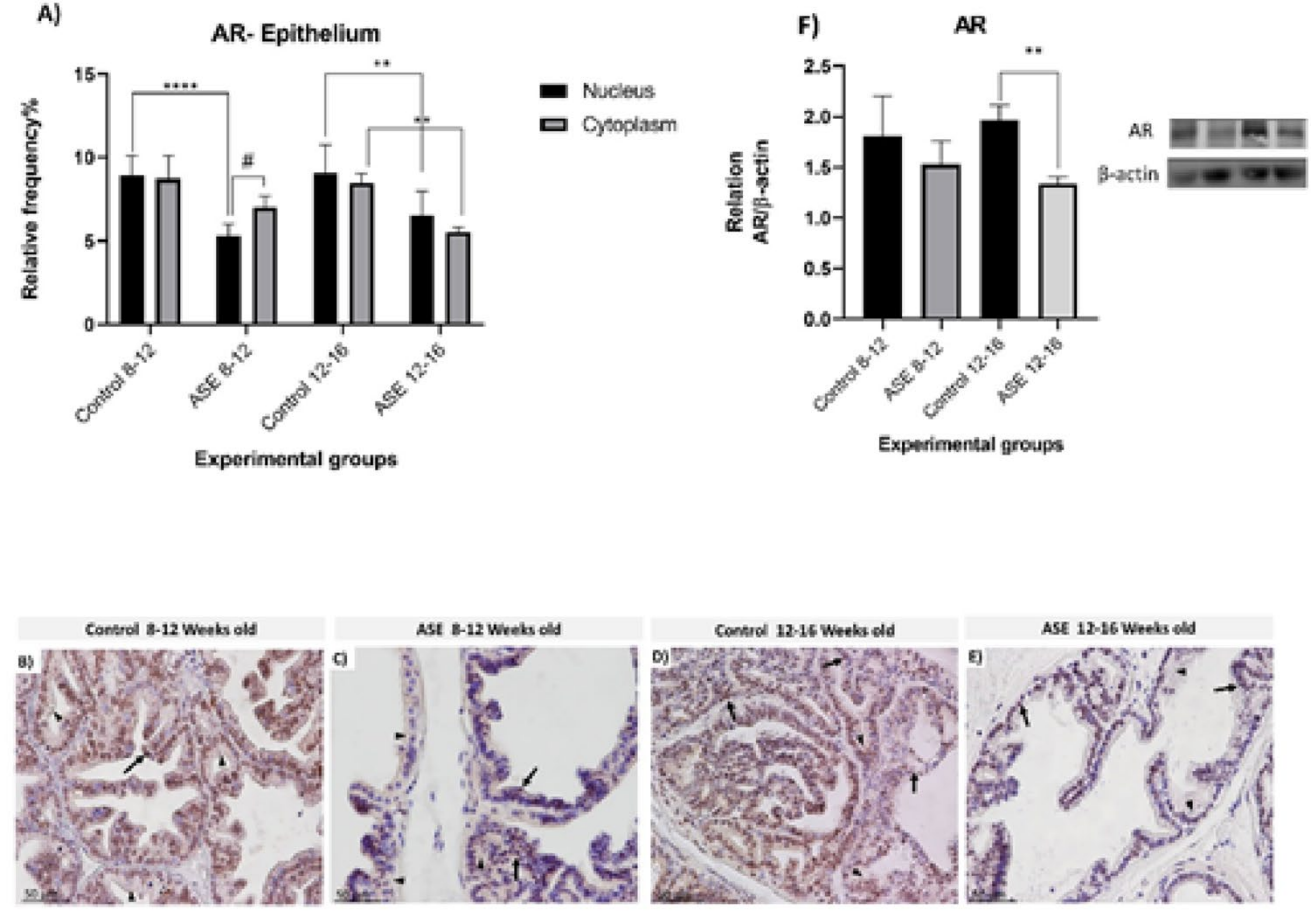
Immunohistochemical analysis and AR protein expression in the dorsolateral prostate lobe of TRAMP mice. **A** Relative frequency (%) of nuclear and cytoplasmic AR immunolabeling in the prostate epithelium of the Control 8–12, ASE 8–12, Control 12–16, and ASE 12–16 groups. Statistical comparisons are indicated by brackets. Asterisks (*) indicate significant differences between Control and ASE groups at the same age, whereas the hash symbol (#) indicates significant differences between nuclear and cytoplasmic AR immunolabeling within the same experimental group. **B**–**E** Representative photomicrographs of AR immunostaining in the prostate epithelium of the experimental groups: **B** Control 8–12 weeks, **C** ASE 8–12 weeks, **D** Control 12–16 weeks, and **E** ASE 12–16 weeks. Images were obtained at 40 × magnification and counterstained with hematoxylin. Arrows indicate positive AR immunostaining. **F** Western blot analysis of AR protein expression in the Control and ASE groups at 8–12 and 12–16 weeks of age, including representative bands of protein blots. Data are presented as mean ± SD. Statistical significance was considered at p < 0.05

ASE treatment reduced AR immunolabeling in both the nucleus and cytoplasm of prostate epithelial cells during the late prostate lesions (including HGPIN and WDAC) (12–16 weeks of age) stages of PCa when compared with their respective control groups (Fig. 5A, D, and E). In the ASE 8–12 group, lower AR immunolabeling was observed in the nucleus compared with the cytoplasm, suggesting greater suppression of functional AR signaling in PCa lesions characterized predominantly by LGPIN compared with lesions containing HGPIN and WDAC (Fig. 5A). Regarding AR protein levels determined by Western blot analysis, no statistically significant differences were observed between the control and ASE-treated groups at 8–12 weeks, whereas a reduction in total AR protein levels was observed in the ASE-treated group at 12–16 weeks compared with its respective control (Fig. 5F).

### ASE induced apoptosis through activation of the intrinsic and extrinsic pathways during PCa progression characterized by HGPIN and WDAC lesions

**Fig. 6:**
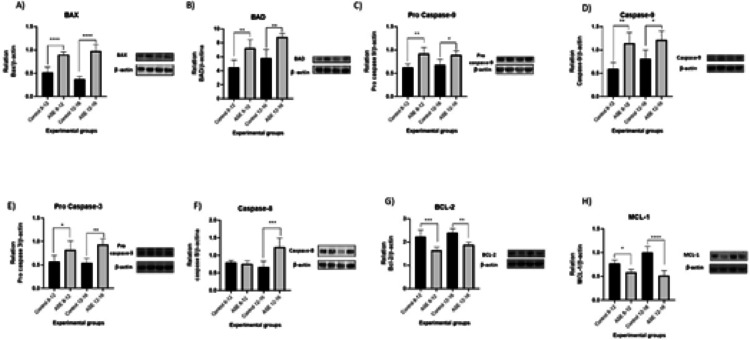
Transgenic adenocarcinoma of the mouse prostate model protein levels at 8–12 and 12–16 weeks old, after ASE treatment. Representative protein blot bands. Statistical significance was considered in the the Control and ASE groups from the same time point (* p < 0.05; ** p < 0.01; *** p < 0.0001)

ASE treatment induced apoptosis in TRAMP mice at different ages. In both the 8–12- and 12–16-week-old groups, ASE increased the levels of pro-apoptotic proteins such as BAX and BAD (Fig. 6A and B).

**Fig. 7:**
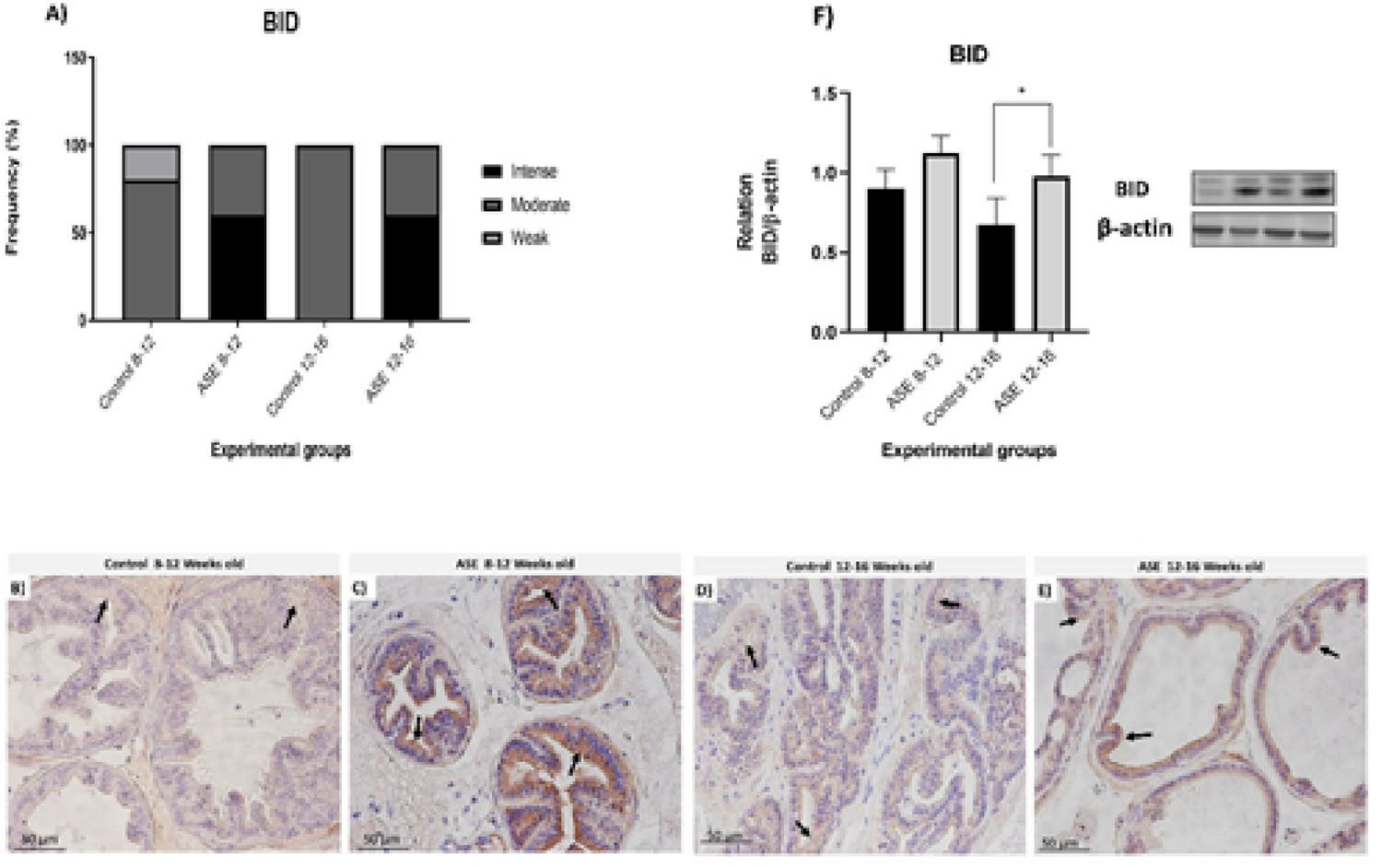
Immunohistochemical analysis and protein expression of BID levels in the dorsolateral lobe of the TRAMP mice prostate. **A** Graph showing the relative frequency of Bid immunostaining in the 8–12- and 12–16-week-old groups. **B**–**E** Representative photomicrographs of cytoplasmic Bid immunostaining in the 8–12- and 12–16-week-old groups: **B** Control 8–12, **C** ASE 8–12, **D** Control 12–16, and **E** ASE 12–16. Magnification: 40 × ; counterstained with hematoxylin. Arrows indicate positive cytoplasmic Bid immunostaining. **F** Western blot analysis showing representative bands of Bid protein expression in the Control and ASE groups at 8–12 and 12–16 weeks of age. Differences between the Control and ASE groups within the same time period were considered statistically significant (*p < 0.05; **p < 0.01)

ASE increased BID protein levels only in animals aged 12–16 weeks (Fig. 7D and H). BID was further evaluated according to the intensity of immunolabeling in the prostate epithelium. In 8–12-week-old mice, moderate to intense immunoreactivity was observed. In the control group, labeling was predominantly moderate (100%), whereas in the ASE-treated group, intense immunolabeling predominated in 60% of the evaluated tissue, and 40% of the animals exhibited moderate labeling (Fig. 7A, B and C). In 12–16-week-old mice, BID immunolabeling ranged from weak to moderate and intense levels. In the control group, weak (20%) and moderate (80%) labeling were observed. In contrast, the ASE-treated group showed a predominance of intense labeling (60%), while the remaining animals exhibited moderate labeling (40%) (Fig. 7A, D and E). Interestingly, BID immunolabeling appeared more pronounced in epithelial regions associated with PIN lesions than in preserved prostatic epithelium, although a specific quantitative comparison between these regions was not performed.

**Fig. 8:**
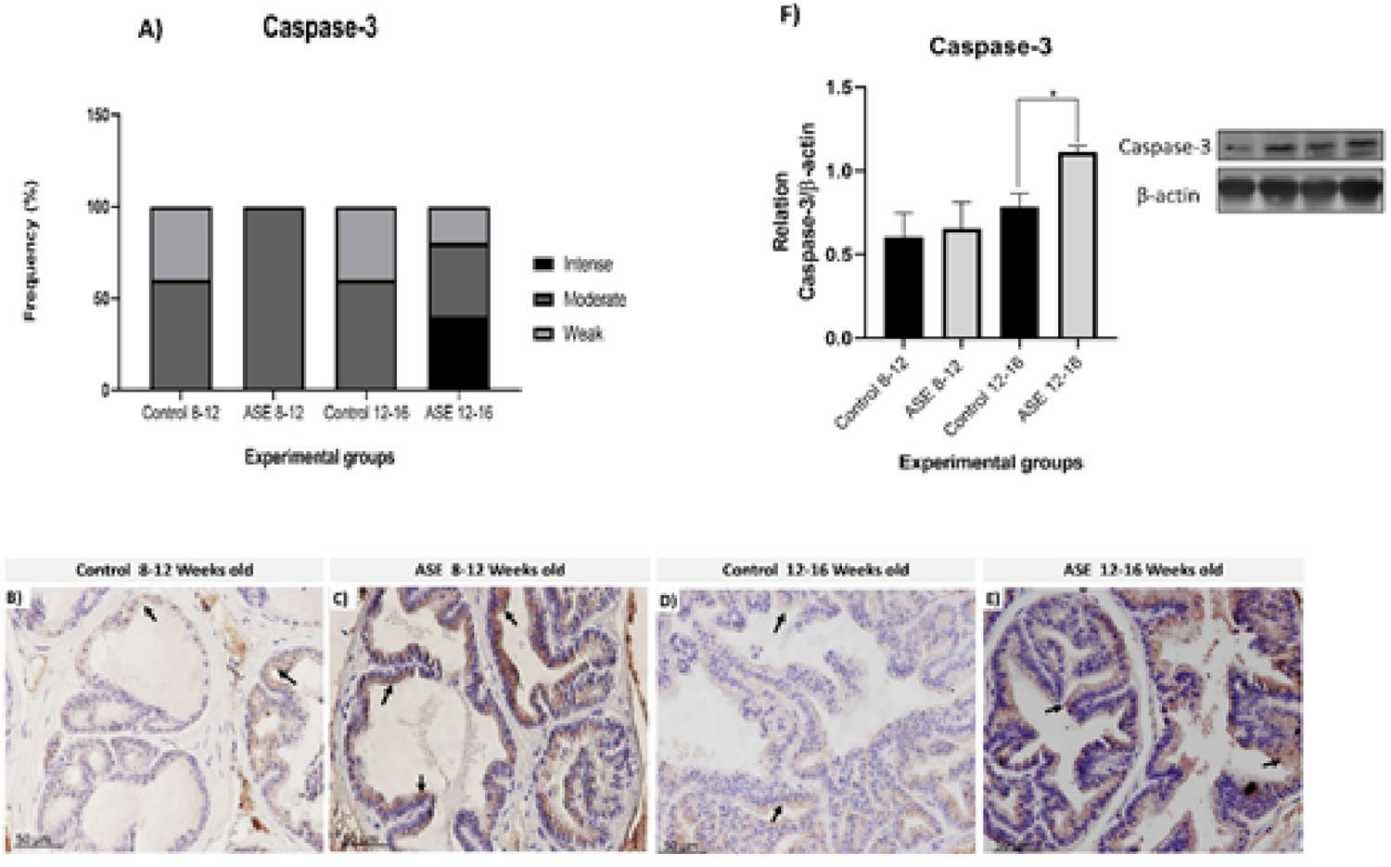
Immunohistochemical analysis and protein expression of Caspase-3 levels in the dorsolateral lobe of the TRAMP mice prostate. **A** Graph showing the relative frequency of Caspase-3 immunostaining in the 8–12- and 12–16-week-old groups. **B**–**E** Representative photomicrographs of cytoplasmic Caspase-3 immunostaining in the 8–12- and 12–16-week-old groups: **B** Control 8–12, **C** ASE 8–12, **D** Control 12–16, and **E** ASE 12–16. Magnification: 40 × ; counterstained with hematoxylin. Arrows indicate positive cytoplasmic Caspase-3 immunostaining. **F** Western blot analysis showing representative bands of Caspase-3 protein expression in the Control and ASE groups at 8–12 and 12–16 weeks of age. Differences between the Control and ASE groups within the same time period were considered statistically significant (*p < 0.05; **p < 0.01)

Regarding caspase activation, increased levels of pro-caspase-9 and caspase-9 proteins were observed in ASE-treated animals compared with controls at both ages (Fig. 6C and D). Pro-caspase-3 protein levels were also increased in ASE-treated animals in both experimental age groups (Fig. 6E). No changes in caspase-3 levels were observed in the ASE 8–12 group; however, a significant increase was detected in the ASE 12–16 group (Fig. 8F). Caspase-3 immunolabeling in the 8–12 control group ranged from weak (40%) to moderate (60%), whereas the ASE 8–12 group showed a predominance of moderate labeling (100%) (Fig. 8A, B and C). In 12–16-week-old mice, the control group exhibited predominantly weak to moderate immunolabeling. In contrast, the ASE-treated group showed increased labeling intensity, with 40% of the animals presenting moderate immunoreactivity, 40% intense labeling, and only 20% weak labeling (Fig. 8A, D and E).

**Fig. 9:**
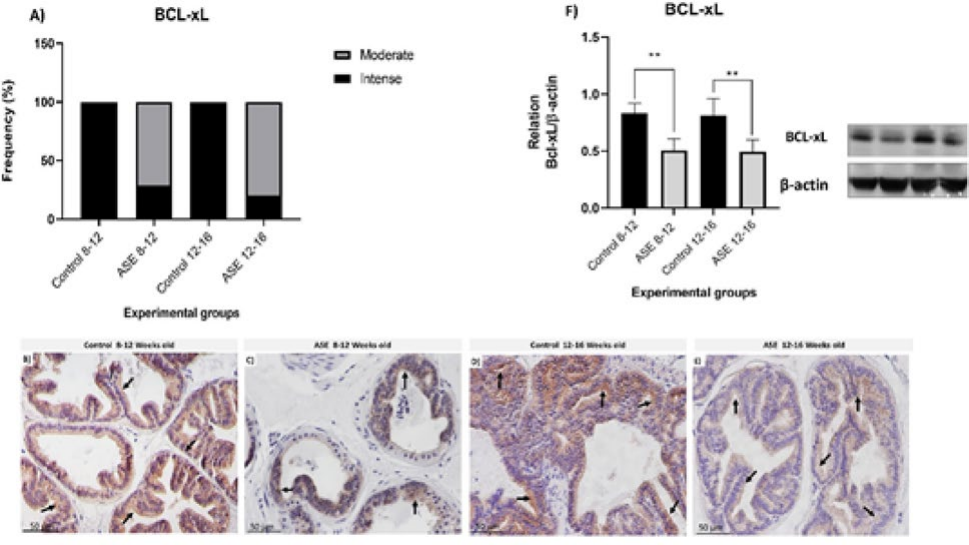
Immunohistochemical analysis and protein expression of BCL-xL levels in the dorsolateral lobe of the TRAMP mice prostate. **A** Graph showing the relative frequency of BCL-xL immunostaining in the 8–12- and 12–16-week-old groups. **B**–**E** Representative photomicrographs of cytoplasmic BCL-xL immunostaining in the 8–12- and 12–16-week-old groups: **B** Control 8–12, (C) ASE 8–12, (D) Control 12–16, and **E** ASE 12–16. Magnification: 40 × ; counterstained with hematoxylin. Arrows indicate positive cytoplasmic BCL-xL immunostaining. **F** Western blot analysis showing representative bands of Bcl-xL protein expression in the Control and ASE groups at 8–12 and 12–16 weeks of age. Differences between the Control and ASE groups within the same time period were considered statistically significant (*p < 0.05; **p < 0.01)

ASE did not affect caspase-8 protein levels in 8–12-week-old mice (Fig. 6F). However, in 12–16-week-old mice, ASE treatment resulted in increased caspase-8 protein levels (Fig. 6F). In contrast, ASE treatment reduced the levels of the anti-apoptotic proteins BCL-2 and MCL-1 in mice, regardless of lesion profiles (Fig. 6G and H). The anti-apoptotic protein BCL-xL was also evaluated in terms of tissue immunolocalization and total protein levels across experimental groups. A decrease in total BCL-xL protein levels was observed in animals treated with ASE at both ages (Fig. 9F). Regarding immunolabeling, the ASE-treated group showed predominantly moderate labeling (70%), whereas the control group exhibited intense labeling (100%). A similar pattern was observed in the ASE 12–16-week-old group (Fig. 9A, B, C, D and E).

## Discussion

Cancer is a complex and multifactorial disease characterized by the transformation of normal cells into malignant cells, a process associated with dysregulated cell proliferation and the progressive accumulation of genetic and molecular alterations. However, tumorigenesis does not depend exclusively on tumor cells. Tumor development and progression are strongly influenced by the tumor microenvironment, which consists of a dynamic network of non-malignant cells, extracellular matrix components, signaling molecules, and stromal interactions that collectively promote tumor maintenance, invasion, and dissemination (Al Hrout et al. 2022). Therapeutic strategies capable of modulating both tumor cells and the surrounding microenvironment have attracted considerable interest in cancer research.

Natural bioactive compounds have emerged as promising chemopreventive and therapeutic agents due to their multitarget biological activities and relatively low toxicity profiles. Several studies have demonstrated that plant-derived compounds may reduce unintended damage to healthy tissues while simultaneously enhancing the efficacy of anticancer therapies (Mohmmed Hegab et al. 2025). Among these compounds, polyphenols stand out because of their broad distribution in fruits, vegetables, teas, nuts, and other plant-derived foods, as well as their wide range of biological activities associated with antioxidant, anti-inflammatory, antiproliferative, and anticancer effects (Hamdy et al. 2026).

Importantly, growing evidence indicates that polyphenols can also regulate epigenetic and post-transcriptional mechanisms involved in tumor progression. Bioactive compounds such as epigallocatechin gallate, genistein, resveratrol, curcumin, and quercetin have been described as modulators of microRNAs (miRNAs) and long non-coding RNAs (lncRNAs) associated with cell proliferation, apoptosis, inflammation, migration, and metastasis in different tumor types, including PCa (Hamdy et al. 2026). Quercetin, one of the major compounds identified in ASE, has been shown to suppress oncogenic lncRNAs such as MALAT1 and UCA1 in PCa cells, thereby reducing epithelial–mesenchymal transition, invasion, migration, and resistance to both apoptosis and chemotherapy (Hamdy et al. 2026).

Among the phenolic compounds present in ASE, gallic acid has attracted considerable attention due to its diverse pharmacological activities. This natural phenolic compound exhibits antioxidant, anti-inflammatory, antimicrobial, cardioprotective, and anticancer properties, reinforcing its potential as a promising bioactive agent for preventing and treating diseases associated with oxidative stress and cellular dysregulation (Ali et al. 2025).

*A. crassiflora* by-products, particularly the seeds, have become the focus of growing scientific interest due to their rich content of bioactive compounds. *A. crassiflora* seeds are known to contain significant levels of phenolic compounds, such as ellagic acid, gallic acid, rutin, and quercetin (Prado et al. 2020; Oliveira et al. 2024). ASE has previously been characterized by our research group, demonstrating high antioxidant capacity as confirmed by three different assays (ABTS, ORAC, and FRAP) (Lemos et al. 2025). Its chemical profile revealed high concentrations of phenolic compounds, including gallic acid, catechin, quercetin, and rutin (Lemos et al. 2025). In addition, acetogenins are found in abundance in this underexplored fraction of *A. crassiflora* (Arruda & Pastore 2019).

Studies investigating the effects of *A. crassiflora* seeds on PCa have been predominantly carried out in cell lines, and therefore the underlying mechanistic pathways remain poorly explored (Prado et al. 2020). Our research group was the first to evaluate the effects of *A. crassiflora* seeds in four PCa cell lines and one non-tumor cell line with distinct morphological characteristics, demonstrating promising pro-apoptotic effects in vitro (Lemos et al. 2025). However, evidence regarding ASE activity remains largely restricted to cell culture models, lacking validation in experimental systems that more closely reproduce prostate carcinogenesis. Thus, the present study demonstrates, for the first time, the pro-apoptotic and antitumoral effects of ASE in a preclinical in vivo model of PCa, specifically in the dorsolateral lobe of TRAMP mice, focusing on the mechanistic pathways involved in apoptosis regulation.

PCa development in TRAMP mice is characterized by progressive morphological changes, maintenance of AR signaling, increased cell proliferation, and, consequently, suppression of apoptosis (Kido et al. 2019). In this model, the dorsolateral lobe of the prostate is particularly relevant for investigation, as it shares important similarities with the peripheral zone of the human prostate, the region in which most neoplasms originate, accounting for more than 60% of cases (Rezende et al. 2025). Research investigating natural compounds derived from plant species using the TRAMP model has increased steadily, demonstrating both chemopreventive and therapeutic effects against PCa (Baseggio et al. 2021). Nevertheless, in vivo studies evaluating the effects of *A. crassiflora* remain scarce.

The present study demonstrates that ASE exerts a protective effect on the prostatic epithelium of the dorsolateral lobe in TRAMP mice during early prostate lesions (predominantly LGPIN) and late prostate lesions (including HGPIN and WDAC) of PCa. Furthermore, ASE slowed the progression of premalignant lesions and reduced the incidence of WDAC, particularly in the ASE 12–16 group. These findings suggest that ASE acts as a modulator of prostate carcinogenesis, possibly through suppression of cell proliferation, an effect supported by the observed decrease in PCNA expression in both experimental age groups.

Supporting these findings, Yang et al. (2015) evaluated an extract from *A. muricata* leaves, a species with phytochemical composition similar to *A. crassiflora*, using a subcutaneous PC-3 xenograft model in male BALB/c nude mice. In that study, extract administration inhibited prostate tumor growth (Yang et al. 2015). Similarly, in TRAMP mice, bitter melon (*Momordica charantia*) extract delayed PCa progression at 21 weeks of age, promoting greater preservation of normal prostatic epithelium and reducing the frequency of high-grade prostatic intraepithelial neoplasia (Ru et al. 2011). Likewise, administration of an isolated polyphenolic fraction from green tea reduced the formation of palpable tumors and inhibited metastasis to lymph nodes, liver, and bone in TRAMP mice, accompanied by decreased PCNA expression (Gupta et al. 2001).

Regarding AR signaling, ASE treatment promoted a reduction in AR levels in both the cytoplasm and nucleus of cells in the dorsolateral lobe of TRAMP mice at early prostate lesions (predominantly LGPIN) and late prostate lesions (including HGPIN and WDAC) of PCa. At early prostate lesions, greater cytoplasmic AR retention was observed in the ASE-treated group compared with nuclear localization, suggesting that ASE may impair AR nuclear translocation. Importantly, despite the altered subcellular distribution of AR observed by immunohistochemistry during the early prostate lesions (predominantly LGPIN), no significant differences were detected in total AR protein levels between the control and ASE-treated groups at 8–12 weeks of age. These findings suggest that alterations in AR subcellular localization may occur during early prostate lesions without proportional changes in total AR protein levels. At the late stage of PCa, a marked overall reduction in AR was detected, with decreased levels in both cellular compartments and reduced protein expression, indicating an effective suppressive effect on this signaling pathway. Collectively, these findings indicate that ASE interferes with AR signaling, a key pathway in prostate carcinogenesis, thereby contributing to disease progression delay in this model.

In TRAMP mice, PCa progression is characterized by an initial androgen-sensitive profile followed by the development of more aggressive lesions associated with androgen independence, closely resembling disease progression in humans (Mishra et al. 2024). Androgens, particularly DHT, bind to AR in the cytoplasm, inducing conformational changes that promote its dissociation from inhibitory proteins (Cai et al. 2018; Chaturvedi and Dehm 2019). Subsequently, the AR–DHT complex translocates to the nucleus, where it binds to specific DNA regions, regulating the transcription of genes involved in prostate cell differentiation and survival (Cai et al. 2018; Chaturvedi & Dehm 2019). Thus, AR expression and activity play a fundamental role in the PCa microenvironment under both normal and neoplastic conditions (Zhou et al. 2015; Aurilio et al. 2020). Accordingly, compounds capable of interfering with AR signaling particularly those capable of reducing or blocking AR activity, are considered promising therapeutic agents for PCa treatment (Demian et al. 2024).

ASE has previously been shown to reduce AR levels in LNCaP and 22Rv1 cells (Lemos et al. 2025). In agreement with these findings, Tummala et al. (2017) demonstrated that quercetin, alone or in combination with enzalutamide, suppressed AR levels in a 22Rv1 xenograft model in SCID mice (Tummala et al. 2017). Given that ASE contains substantial amounts of quercetin, as reported by our research group (Lemos et al. 2025), this compound likely contributes significantly to AR suppression observed in PCa. AR activation directly influences cell proliferation and may also interact with cell death–related signaling pathways to regulate tumor progression (Wang et al. 2014). Accordingly, AR plays a complex role in cell death mechanisms, including apoptosis, necrosis, and autophagy (Wen et al., 2014). Notably, AR exhibits a dual role in apoptosis regulation, with the capacity to either promote or inhibit apoptotic processes depending on microenvironmental conditions and cellular context (Wen et al., 2014).

Apoptosis is a programmed cell death process essential for tissue homeostasis and cancer suppression, and its dysregulation contributes to tumor development and progression (Morana et al. 2022). Apoptotic signaling occurs mainly through two interconnected pathways: the extrinsic (death receptor-mediated) pathway and the intrinsic (mitochondrial) pathway (Yaacoub et al. 2016). In the present study, ASE modulated proteins associated with both pathways, suggesting a coordinated pro-apoptotic effect during PCa progression.

The extrinsic pathway is initiated through activation of death receptors and subsequent recruitment of the adaptor protein FADD, leading to activation of caspase-8 and downstream effector caspases responsible for apoptotic execution (Yaacoub et al. 2016). In the present study, ASE positively regulated caspase-8 in late prostate lesions (including HGPIN and WDAC) of PCa, concomitant with activation of procaspase-3 and caspase-3, suggesting activation of the extrinsic apoptotic pathway at advanced stages of tumor progression. In contrast, caspase-8 levels remained unchanged during early prostate lesions (predominantly LGPIN), which may explain the absence of caspase-3 activation at this stage.

Similar findings were reported by Lemos et al. (2025), in which ASE increased caspase-8 and procaspase-3 levels in androgen-dependent LNCaP cells, whereas no alterations were observed in androgen-independent PC-3 and 22Rv1 cells. Likewise, crude *A. crassiflora* extract did not affect caspase-8 expression in SiHa cells (Silva et al. 2018). Caspase-8 is synthesized as an inactive proenzyme that becomes activated following oligomerization within death receptor signaling complexes (Fulda 2009). Once activated, caspase-8 cleaves and activates caspase-3, thereby promoting apoptotic execution (Tummers and Green 2017).

Importantly, caspase-8 also represents an essential molecular link between the extrinsic and intrinsic pathways through BID cleavage (Tummers and Green 2017). Upon cleavage, truncated BID translocates to mitochondria and promotes BAX/BAK-mediated mitochondrial membrane permeabilization, amplifying apoptotic signaling (Qian et al. 2022; Wyżewski et al. 2025). Therefore, modulation of BID may contribute to coordinated activation of mitochondrial apoptosis by ASE.

The intrinsic apoptotic pathway is activated in response to intracellular stress signals such as oxidative stress and DNA damage and is tightly regulated by proteins of the Bcl-2 family (Yaacoub et al. 2016; Kashyap et al. 2021). In this pathway, the balance between pro-apoptotic and anti-apoptotic proteins determines mitochondrial outer membrane permeabilization and subsequent activation of downstream caspases (Yuan and Ofengeim 2024).

In the present study, ASE upregulated the pro-apoptotic proteins BAD, BID, and BAX regardless of PCa stage, while simultaneously downregulating the anti-apoptotic proteins BCL-2, BCL-xL, and MCL-1. These findings indicate that ASE promotes a favorable pro-apoptotic balance that may facilitate mitochondrial dysfunction and apoptotic progression.

BID, BAD, and BAX exert complementary but distinct functions during intrinsic apoptosis. Following activation, BID cooperates with BAX by facilitating its insertion into the mitochondrial membrane, thereby enhancing mitochondrial permeabilization (Qian et al. 2022). BAD contributes to apoptosis by neutralizing anti-apoptotic proteins such as BCL-2 and BCL-xL, allowing BAX activation and cytochrome c release (Bui et al. 2018; Wu et al. 2021). BAX and BAK activation represent a critical irreversible step in intrinsic apoptosis, leading to mitochondrial dysfunction and release of apoptogenic factors (Li et al. 2021).

Previous studies involving *A. crassiflora*-derived compounds and related phytochemicals are in agreement with these findings. Lemos et al. (2025) demonstrated increased BAX expression in LNCaP and 22Rv1 cells treated with ASE. Quercetin, identified as a major compound in ASE, increased BAX levels in PC-3 and LNCaP xenograft tumors and promoted BAD activation in LNCaP cells (Lee et al. 2008; Yang et al. 2015). Similarly, quercetin-3-glycoside induced BID cleavage and increased BAX levels in HeLa cells (Nile et al. 2021), whereas *A. muricata* extracts increased BAX expression and mitochondrial disruption in colon cancer cells (Zorofchian Moghadamtousi et al. 2014).

Mitochondrial permeabilization promotes cytochrome c release and apoptosome formation with Apaf-1, leading to activation of initiator caspase-9 and downstream effector caspase-3 (Asadi et al. 2022). In agreement with this mechanism, ASE increased initiator and effector caspase levels in late prostate lesions (including HGPIN and WDAC) of PCa, whereas caspase-3 activation was not observed in early prostate lesions (predominantly LGPIN), suggesting stage-dependent apoptotic activation.

In agreement with the present findings, Lemos et al. (2025) reported activation of procaspase-9, caspase-9, and procaspase-3 in LNCaP and 22Rv1 cells treated with ASE. Likewise, crude *A. crassiflora* extract activated caspase-3 and caspase-9 in cervical cancer models (Silva et al. 2018). Catechin, another major ASE compound, also activated caspase-9 and caspase-3 in PC-3 cells (Chen and Tsai 2016). Furthermore, quercetin alone or combined with resveratrol promoted caspase-8 and caspase-9 cleavage in aged TRAMP mice (Singh et al. 2020).

Anti-apoptotic proteins such as BCL-2, BCL-xL, and MCL-1 are critical regulators of mitochondrial apoptosis and contribute to tumor progression and therapeutic resistance by preventing BAX/BAK activation and cytochrome c release (Tzifi et al. 2011; Valentini et al. 2022). ASE reduced BCL-2, BCL-xL, and MCL-1 protein levels across different lesion profiles of PCa, reinforcing its potential as a pro-apoptotic agent.

These findings are supported by previous studies demonstrating that ASE reduced BCL-2 and BCL-xL expression in androgen-dependent and androgen-independent PCa cell lines (Lemos et al. 2025). In addition, quercetin combined with resveratrol reduced BCL-2 levels in TRAMP mice (Singh et al. 2020), while ferulic acid downregulated BCL-2 and MCL-1 expression in cervical carcinoma models (Luo et al. 2020). Collectively, these findings reinforce the antitumor potential of ASE and related phytochemicals through coordinated modulation of apoptotic regulators.

**Fig. 10:**
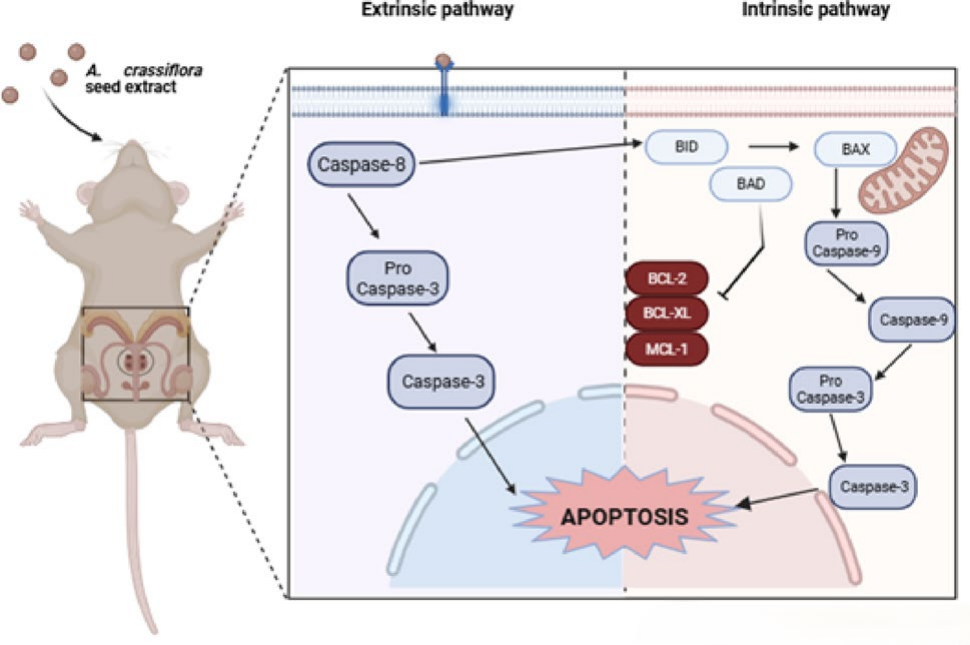
Schematic representation of apoptosis induction by extrinsic and intrinsic pathways in late prostate cancer. There is an increase in caspase-8, responsible for the activation of Bid and consequent integration between the two pathways, as well as an increase in Bad, Bax, caspase-9, and caspase-3, indicating sequential activation of the apoptotic cascade. In contrast, there is a reduction in the antiapoptotic proteins Bcl-2, Bcl-xL, and MCL-1, favoring mitochondrial permeabilization and the progression of programmed cell death. Illustration created using BioRender.com

Overall, the present findings suggest that ASE promotes apoptosis in PCa through coordinated modulation of both extrinsic and intrinsic apoptotic pathways (Fig. 10). The simultaneous activation of pro-apoptotic mediators and suppression of anti-apoptotic proteins may contribute to mitochondrial dysfunction, caspase activation, and apoptotic execution, supporting the therapeutic potential of ASE against PCa progression. Despite these findings, the literature remains limited regarding the biological effects of *A. crassiflora*, particularly within the PCa microenvironment, underscoring the need for further investigation of this native Brazilian fruit.

In addition to its antitumoral effects, the safety profile of ASE is a critical aspect for its potential application. Therefore, in the present study, toxicity parameters were evaluated, including body weight and hepatic histopathology of the animals. According to Josephat et al. (2024), a reduction in body weight may be associated with toxic effects of plant-derived compounds, which can lead to decreased appetite and, consequently, reduced caloric intake. However, in the present study, a gradual increase in body weight was observed in both the control group (water containing 10% DMSO) and the ASE treated group throughout the experimental period. The absence of these alterations in the present study suggests that neither the extract nor the dilution vehicle compromised normal metabolism or feeding regulation, which, when affected, typically result in reduced food intake and body weight loss (Josephat et al. 2024).

Histopathological evaluation of organs is widely recognized as an essential parameter for determining the safety of experimental substances, especially in in vivo studies (Al-Afifi et al. 2018). Taking this aspect into consideration, the liver plays a central role, as it is the primary organ involved in the metabolism of potentially toxic compounds ingested through the diet (Josephat et al. 2024). In the present study, the preservation of hepatic histological architecture, without relevant morphological alterations in hepatocytes, reinforces the absence of toxic effects associated with the treatment, corroborating the body weight findings.

Despite the promising findings of the present study, some limitations should be considered. The in vivo experiments were conducted using only a single ASE concentration. Although the chosen dose was supported by preliminary pilot experiments conducted by our research group as well as by previous studies involving Annonaceae-derived compounds, further pharmacokinetic and toxicological characterization is still necessary. Future investigations could incorporate multiple dose levels, standard therapeutic controls, and complementary mechanistic analyses to better characterize the therapeutic potential, safety profile, and translational relevance of ASE during PCa progression.

Another point that could be a limitation aspect in the present study would be the absence of a validated positive control, such as Docetaxel or Enzalutamide, which could provide a benchmark for comparative efficacy and translational relevance. However, this study was designed as an initial preclinical investigation aimed primarily at evaluating, for the first time, the biological and mechanistic effects of ASE during PCa progression in the TRAMP model. Given the limited literature regarding the biological effects of *A. crassiflora*, particularly in PCa, our initial objective was to establish whether ASE could exert antitumoral activity and modulate apoptosis-related pathways in vivo before progressing to comparative therapeutic approaches involving conventional anticancer drugs. Nevertheless, future investigations should incorporate standard anticancer therapies, both alone and in combination with ASE, to better determine potential synergistic effects, translational applicability, and comparative efficacy relative to clinically established treatments.

Another aspect to mention is related to the TRAMP model and some limitation as an animal model, considering that each animal model has inherent limitations. Although the TRAMP model is widely recognized as an important preclinical platform for investigating prostate carcinogenesis progression and chemopreventive interventions, due to its reproducible development of lesions ranging from PIN to adenocarcinoma, this model does not fully recapitulate all aspects of human prostate cancer biology (Kido et al. 2019). One of the major limitations associated with murine PCa models is the relatively low incidence and progression of bone metastasis compared with human disease, as well as the high frequency of neuroendocrine adenocarcinoma phenotypes, which represent only a small proportion of PCa cases in humans (Chiaverotti et al. 2008; Rickman et al. 2017; Kido et al. 2019; Saranyutanon et al. 2020). In addition, TRAMP tumors may progress toward neuroendocrine phenotypes associated with SV40 T antigen-mediated inactivation of the p53 and Rb pathways, a characteristic that differs from the most prevalent forms of human prostate adenocarcinoma (Chiaverotti et al. 2008; Rickman et al. 2017; Kido et al. 2019; Saranyutanon et al. 2020). Nevertheless, despite these limitations, the TRAMP model remains a valuable and well-established experimental tool for investigating PCa progression, androgen signaling, and the chemopreventive potential of food-derived bioactive compounds capable of delaying PCa progression (Berman-booty et al. 2012; Kido et al. 2019).

## Conclusion

The results of this study highlight the promising effects of ASE against PCa progression. ASE modulated key biological processes involved in prostate carcinogenesis progression, particularly in late prostate lesions (including HGPIN and WDAC) of PCa. In addition, ASE did not induce hepatotoxicity, corroborating its safety profile in a preclinical model. The maintenance of healthy prostatic epithelium and the delay in the progression of premalignant and malignant lesions were observed in TRAMP mice and were associated with decreased cell proliferation, suppression of androgenic activity, and activation of apoptosis. ASE was shown to suppression of AR signaling, modulating a central signaling pathway for tumor survival and progression. Furthermore, ASE demonstrated, for the first time, its ability to induce apoptosis through different pathways in late prostate lesions (including HGPIN and WDAC) of PCa in vivo. ASE favors a pro-apoptotic balance by increasing BAX expression and reducing BCL-2 expression, key regulators of mitochondrial permeabilization, in addition to increasing active caspase-3 levels, an enzyme indispensable in both the intrinsic and extrinsic pathways. Thus, ASE promotes a pro-apoptotic environment unfavorable to tumor survival. Taken together, these results reinforce the potential of ASE to modulate prostatic homeostasis, indicating a promising strategy with chemopreventive effects and potential as an adjuvant therapy against prostate cancer.

## Supplementary Information

Below is the link to the electronic supplementary material.

Supplementary Material 1

## Data Availability

The data that support the findings of this study are not openly available due to reasons of sensitivity and are available from the corresponding author upon reasonable request.

## Abbreviations

ABTS: 2,2′-Azinobis-(3-ethylbenzothiazoline-6-sulfonic acid)
Apaf-1: Apoptotic protease activating factor-1
AR: Androgen receptor
ASE: *Annona crassiflora* Seed extract
BAD: BCL2-associated death agonist
BAK: BCL-2 antagonist killer
BAX: BCL-2-associated X protein
BCL-2: B-cell lymphoma 2
Bcl-xL: B-cell lymphoma-extra-large
BID: BH3-interacting domain death agonist
DHT: Dihydrotestosterone
DMSO: Dimethyl sulfoxide
FADD: Fas-associated death domain protein
FRAP: Ferric Reducing Antioxidant Power
HGPIN: High-grade prostatic intraepithelial neoplasia
LGPIN: Low-grade prostatic intraepithelial neoplasia
MCL-1: Myeloid cell leukemia-1
ORAC: Oxygen Radical Absorbance Capacity
PCa: Prostate cancer
PCNA: Proliferating cell nuclear antigen
TRAMP: Transgenic Adenocarcinoma of the Mouse Prostate
WDAC: Well-differentiated adenocarcinoma

## References

[CR1] Ahmad N, Qamar M, Yuan Y et al (2022) Dietary polyphenols: extraction, identification, bioavailability, and role for prevention and treatment of colorectal and prostate cancers. Molecules 27:2831. 10.3390/molecules27092831 35566182 10.3390/molecules27092831 PMC9104150

[CR2] Al-Afifi NA, Alabsi AM, Bakri MM, Ramanathan A (2018) Acute and sub-acute oral toxicity of Dracaena cinnabari resin methanol extract in rats. BMC Complement Altern Med 18:50. 10.1186/s12906-018-2110-3 29402248 10.1186/s12906-018-2110-3 PMC5800047

[CR3] Al Hrout A, Cervantes-Gracia K, Chahwan R, Amin A (2022) Modelling liver cancer microenvironment using a novel 3D culture system. Sci Rep 12:8003. 10.1038/s41598-022-11641-7 35568708 10.1038/s41598-022-11641-7 PMC9107483

[CR4] Ali MAM, Matouk AI, Hamza AA et al (2025) Gallic and glycyrrhetinic acids prevent azithromycin-induced liver damage in rats by mitigating oxidative stress and inflammation. Sci Rep 15:9566. 10.1038/s41598-025-93120-3 40113827 10.1038/s41598-025-93120-3 PMC11926359

[CR5] Arruda HS, Pastore GM (2019) Araticum (Annona crassiflora Mart.) as a source of nutrients and bioactive compounds for food and non-food purposes: a comprehensive review. Food Res Int 123:450–480. 10.1016/j.foodres.2019.05.011 31284996 10.1016/j.foodres.2019.05.011

[CR6] Arruda HS, Pereira GA, Pastore GM (2018) Brazilian Cerrado fruit araticum (Annona crassiflora Mart.) as a potential source of natural antioxidant compounds. Int Food Res J 25:2005–2012

[CR7] Asadi M, Taghizadeh S, Kaviani E et al (2022) Caspase-3: structure, function, and biotechnological aspects. Biotechnol Appl Biochem 69:1633–1645. 10.1002/bab.2233 34342377 10.1002/bab.2233

[CR8] Aurilio G, Cimadamore A, Mazzucchelli R et al (2020) Androgen receptor signaling pathway in prostate cancer: from genetics to clinical applications. Cells 9:2653. 10.3390/cells9122653 33321757 10.3390/cells9122653 PMC7763510

[CR9] Awad B, Hamza AA, Al-Maktoum A et al (2023) Combining crocin and sorafenib improves their tumor-inhibiting effects in a rat model of diethylnitrosamine-induced cirrhotic-hepatocellular carcinoma. Cancers (Basel) 15:4063. 10.3390/cancers15164063 37627094 10.3390/cancers15164063 PMC10452334

[CR10] Basak D, Gregori L, Johora F, Deb S (2022) Preclinical and clinical research models of prostate cancer: a brief overview. Life 12:1607. 10.3390/life12101607 36295041 10.3390/life12101607 PMC9605520

[CR11] Baseggio AM, Kido LA, Viganó J et al (2021) Systemic antioxidant and anti-inflammatory effects of yellow passion fruit bagasse extract during prostate cancer progression. J Food Biochem 46:1–16. 10.1111/jfbc.13885 10.1111/jfbc.13885 34338308

[CR12] Bergengren O, Pekala KR, Matsoukas K et al (2023) 2022 Update on prostate cancer epidemiology and risk factors—a systematic review. Eur Urol 84:191–206. 10.1016/j.eururo.2023.04.021 37202314 10.1016/j.eururo.2023.04.021 PMC10851915

[CR13] Berman-booty LD, Sargeant AM, Rosol TJ, Rengel RC (2012) NIH Public Access. Toxicol Pathol 40:5–17. 10.1177/0192623311425062 22021166 10.1177/0192623311425062 PMC4271830

[CR14] Bouabdallah S, Brinza I, Boiangiu RS et al (2024) The effect of a tribulus-based formulation in alleviating cholinergic system impairment and scopolamine-induced memory loss in zebrafish (danio rerio): Insights from molecular docking and in vitro/in vivo approaches. Pharmaceuticals 17:200. 10.3390/ph17020200 38399415 10.3390/ph17020200 PMC10891926

[CR15] Bui N-L-C, Pandey V, Zhu T et al (2018) Bad phosphorylation as a target of inhibition in oncology. Cancer Lett 415:177–186. 10.1016/j.canlet.2017.11.017 29175460 10.1016/j.canlet.2017.11.017

[CR16] Cai Z, Chen W, Zhang J, Li H (2018) Androgen receptor: what we know and what we expect in castration-resistant prostate cancer. Int Urol Nephrol 50:1753–1764. 10.1007/s11255-018-1964-0 30128923 10.1007/s11255-018-1964-0

[CR17] Chaturvedi AP, Dehm SM (2019) Androgen Receptor Dependence. In: Prostate Cancer Cellular and Genetic Mechanisms of Disease Development and Progression. 185–237

[CR18] Chen B-H, Tsai YJ (2016) Preparation of catechin extracts and nanoemulsions from green tea leaf waste and their inhibition effect on prostate cancer cell PC-3. Int J Nanomedicine 11:1907. 10.2147/IJN.S103759 27226712 10.2147/IJN.S103759 PMC4866752

[CR19] Chiaverotti T, Couto SS, Donjacour A et al (2008) Dissociation of epithelial and neuroendocrine carcinoma lineages in the transgenic adenocarcinoma of mouse prostate model of prostate cancer. Am J Pathol 172:236–246. 10.2353/ajpath.2008.070602 18156212 10.2353/ajpath.2008.070602 PMC2189611

[CR20] Colucci M, Maione F, Bonito MC et al (2008) New insights of dimethyl sulphoxide effects (DMSO) on experimental in vivo models of nociception and inflammation. Pharmacol Res 57:419–425. 10.1016/j.phrs.2008.04.004 18508278 10.1016/j.phrs.2008.04.004

[CR21] Costa CAR da, o Nascimento SV, Valadares RB da S, et al (2025) Proteome and metabolome of Annona crassiflora Mart. fruit and their interaction during development. Sci Hortic (Amsterdam). 339: 113809. 10.1016/j.scienta.2024.113809

[CR22] Demian MD, Amasiorah VI, Johnson TO, Ebenyi LN (2024) Phytochemical identification and in silico elucidation of interactions of bioactive compounds from Citrullus lanatus with androgen receptor towards prostate cancer treatment. Silico Pharmacol 12:27. 10.1007/s40203-024-00193-5 10.1007/s40203-024-00193-5 PMC10999405 38596366

[CR23] Ebenyi LN, Chigozie VU, Destiny D, Anyanwu CB (2024) Antioxidative, anti-androgenic, and inhibitory activities of ethanolic extract of Annona muricata leaf on sex hormones-induced benign prostate hyperplasia through in vivo and in silico studies. Nat Prod Res. 10.1080/14786419.2024.2409384 39340243 10.1080/14786419.2024.2409384

[CR24] Emiru AY, Makonnen E, Regassa F et al (2021) Antitrypanosomal activity of hydromethanol extract of leaves of Cymbopogon citratus and seeds of Lepidium sativum: in-vivo mice model. BMC Complement Med Ther 21:290. 10.1186/s12906-021-03449-1 34837971 10.1186/s12906-021-03449-1 PMC8627079

[CR25] Fulda S (2009) Caspase-8 in cancer biology and therapy. Cancer Lett 281:128–133. 10.1016/j.canlet.2008.11.023 19111387 10.1016/j.canlet.2008.11.023

[CR26] Gingrich JR, Barrios RJ, Foster BA, Greenberg NM (1999) Pathologic progression of autochthonous prostate cancer in the TRAMP model. Prostate Cancer Prostatic Dis 2:70–75. 10.1038/sj/pcan/4500296 12496841 10.1038/sj.pcan.4500296

[CR27] Gingrich JR, Greenberg NM (1996) A Transgenic Mouse Prostate Cancer Model. Toxicol Pathol 24:502–504. 10.1177/019262339602400414 8864193 10.1177/019262339602400414

[CR28] Greenberg NM, DeMayo F, Finegold MJ et al (1995) Prostate cancer in a transgenic mouse. Proc Natl Acad Sci 92:3439–3443. 10.1073/pnas.92.8.3439 7724580 10.1073/pnas.92.8.3439 PMC42182

[CR29] Gupta J, Abdulsahib WK, Turki Jalil A et al (2023) Prostate cancer and microRNAs: New insights into apoptosis. Pathol - Res Pract 245:154436. 10.1016/j.prp.2023.154436 37062208 10.1016/j.prp.2023.154436

[CR30] Gupta S, Hastak K, Ahmad N et al (2001) Inhibition of prostate carcinogenesis in TRAMP mice by oral infusion of green tea polyphenols. Proc Natl Acad Sci 98:10350–10355. 10.1073/pnas.171326098 11504910 10.1073/pnas.171326098 PMC56964

[CR31] Hamdy NM, Amin A, Abd-ellatef GEF, et al (2026) An Overview of Targeting Some Cancer Hallmarks with Plant Polyphenols: A Step Toward Precision. 423–477 10.1007/978-3-032-08530-6_8 41479043

[CR32] Hamza AA, Heeba GH, Hassanin SO et al (2023) Hibiscus-cisplatin combination treatment decreases liver toxicity in rats while increasing toxicity in lung cancer cells via oxidative stress- apoptosis pathway. Biomed Pharmacother 165:115148. 10.1016/j.biopha.2023.115148 37450997 10.1016/j.biopha.2023.115148

[CR33] Ittmann M, Huang J, Radaelli E et al (2013) Animal models of human prostate cancer: the consensus report of the New York meeting of the mouse models of human cancers consortium prostate pathology committee. Cancer Res 73:2718–2736. 10.1158/0008-5472.CAN-12-4213 23610450 10.1158/0008-5472.CAN-12-4213 PMC3644021

[CR34] Josephat JK, Mpinda CB, Masalu RJ (2024) Phytochemical profiling and acute oral toxicity of Suregada zanzibariensis ( Baill ) root extract. Afr Health Sci. 10.4314/ahs.v24i2.15 40809565 10.4314/ahs.v24i2.15 PMC12341169

[CR35] Justino AB, Florentino RM, França A et al (2021) Alkaloid and acetogenin-rich fraction from Annona crassiflora fruit peel inhibits proliferation and migration of human liver cancer HepG2 cells. PLoS ONE 16:1–21. 10.1371/journal.pone.0250394 10.1371/journal.pone.0250394 PMC8266062 34237060

[CR36] Kaplan-Lefko PJ, Chen T, Ittmann MM et al (2003) Pathobiology of autochthonous prostate cancer in a pre-clinical transgenic mouse model. Prostate 55:219–237. 10.1002/pros.10215 12692788 10.1002/pros.10215

[CR37] Kashyap D, Garg VK, Goel N (2021) Intrinsic and extrinsic pathways of apoptosis: Role in cancer development and prognosis. In: Advances in Protein Chemistry and Structural Biology, 1st edn. Elsevier Inc., 73–120 10.1016/bs.apcsb.2021.01.003 33931145

[CR38] Kido LA, de Almeida Lamas C, Maróstica MR, Cagnon VHA (2019) Transgenic Adenocarcinoma of the Mouse Prostate (TRAMP) model: A good alternative to study PCa progression and chemoprevention approaches. Life Sci 217:141–147. 10.1016/j.lfs.2018.12.002 30528182 10.1016/j.lfs.2018.12.002

[CR39] Kido LA, Montico F, Sauce R et al (2016) Anti-inflammatory therapies in TRAMP mice: delay in PCa progression. Endocr Relat Cancer 23:235–250. 10.1530/ERC-15-0540 26772819 10.1530/ERC-15-0540

[CR40] Kulac I, Roudier MP, Haffner MC (2024) Molecular Pathology of Prostate Cancer. Clin Lab Med 44:161–180. 10.1016/j.cll.2023.08.003 38821639 10.1016/j.cll.2023.08.003

[CR41] Lee D-H, Szczepanski M, Lee YJ (2008) Role of Bax in quercetin-induced apoptosis in human prostate cancer cells. Biochem Pharmacol 75:2345–2355. 10.1016/j.bcp.2008.03.013 18455702 10.1016/j.bcp.2008.03.013 PMC3266687

[CR42] Lemos IL, Macedo MJ, Santos FR et al (2025) Araticum (Annona crassiflora Mart) by-products suppress cell proliferation and induce apoptosis particularly in androgen-dependent prostate cancer cell lines. Food Res Int. 10.1016/j.foodres.2025.116124 40263819 10.1016/j.foodres.2025.116124

[CR43] Li K, van Delft MF, Dewson G. (2021). Too much death can kill you: inhibiting intrinsic apoptosis to treat disease. EMBO J. 10.15252/embj.2020107341 10.15252/embj.2020107341 PMC8280825 34037273

[CR44] Luo L, Zhu S, Tong Y, Peng S (2020) Ferulic Acid Induces Apoptosis of HeLa and Caski Cervical Carcinoma Cells by Down-Regulating the Phosphatidylinositol 3-Kinase (PI3K)/Akt Signaling Pathway. Med Sci Monit. 10.12659/MSM.920095 10.12659/MSM.920095 PMC7003662 31983729

[CR45] Mishra J, Chakraborty S, Nandi P, et al (2024) Epigenetic regulation of androgen dependent and independent prostate cancer. In: Advances in Cancer Research. Elsevier, 223–320 10.1016/bs.acr.2024.05.007 39032951

[CR46] Mohmmed Hegab AM, Hassanin SO, Mekky RH et al (2025) Withania somnifera ameliorates doxorubicin-induced nephrotoxicity and potentiates its therapeutic efficacy targeting SIRT1/Nrf2, oxidative stress, inflammation, and apoptosis. Pharmaceuticals 18:248. 10.3390/ph18020248 40006061 10.3390/ph18020248 PMC11859695

[CR47] Montgomery DC (1991) Design and Analysis of Experiments, 3rd edn. New York

[CR48] Montico F, Lamas CDA, Rossetto IMU, Baseggio AM, Cagnon VHA (2023) Lobe-specific responses of TRAMP mice dorsolateral prostate following celecoxib and nintedanib therapy. J Mol Histol 54(4):379–403 37335420 10.1007/s10735-023-10130-z

[CR49] Morana O, Wood W, Gregory CD (2022) The apoptosis paradox in cancer. Int J Mol Sci 23:1328. 10.3390/ijms23031328 35163253 10.3390/ijms23031328 PMC8836235

[CR50] Nasir B, Baig MW, Majid M et al (2020) Preclinical anticancer studies on the ethyl acetate leaf extracts of *Datura stramonium* and *Datura inoxia*. BMC Complement Med Ther 20:188. 10.1186/s12906-020-02975-8 32552791 10.1186/s12906-020-02975-8 PMC7302377

[CR51] Nelson DR, Hrout AA, Alzahmi AS et al (2022) Molecular mechanisms behind safranal’s toxicity to HepG2 cells from dual omics. Antioxidants 11:1125. 10.3390/antiox11061125 35740022 10.3390/antiox11061125 PMC9219844

[CR52] Nile A, Nile SH, Shin J et al (2021) Quercetin-3-glucoside extracted from apple pomace induces cell cycle arrest and apoptosis by increasing intracellular ROS levels. Int J Mol Sci 22:10749. 10.3390/ijms221910749 34639090 10.3390/ijms221910749 PMC8509831

[CR53] Nogueira Pangrazi E, da Silva RF, Kido LA et al (2018) Nintedanib treatment delays prostate dorsolateral lobe cancer progression in the TRAMP model: contribution to the epithelial-stromal interaction balance. Cell Biol Int 42:153–168. 10.1002/cbin.10881 28980742 10.1002/cbin.10881

[CR54] de Oliveira PF, Felix Ávila P, de Melo Carolo dos Santos M, et al (2024) Antioxidant, antimutagenic, and hypoglycemic properties of flours by different parts of marolo (Annona crassiflora Mart) seeds: film and almond. Food Res Int 196:115055. 10.1016/j.foodres.2024.115055 39614560 10.1016/j.foodres.2024.115055

[CR55] Prado LG, Arruda HS, Peixoto Araujo NM et al (2020) Antioxidant, antiproliferative and healing properties of araticum (Annona crassiflora Mart) peel and seed. Food Res Int. 10.1016/j.foodres.2020.109168 32466931 10.1016/j.foodres.2020.109168

[CR56] Qian S, Wei Z, Yang W, Yang Y et al (2022) The role of BCL-2 family proteins in regulating apoptosis and cancer therapy. Front Oncol 12:1–16. 10.3389/fonc.2022.985363 10.3389/fonc.2022.985363 PMC9597512 36313628

[CR57] Rezende BB, Vecchi ACT, Maróstica MR et al (2025) Differential effects of jaboticaba peel extract administration on PCa progression in TRAMP mice depend on the androgenic status of the prostatic milieu and are driven by angiogenesis regulation. Food Res Int 2008:116155. 10.1016/j.foodres.2025.116155 10.1016/j.foodres.2025.116155 40263783

[CR58] Rickman DS, Beltran H, Demichelis F, Rubin MA (2017) Biology and evolution of poorly differentiated neuroendocrine tumors. Nat Med 23:664–673. 10.1038/nm.4341 10.1038/nm.4341 28586335

[CR59] Rosa MN, e Silva LR V., Longato GB, et al (2021) Bioprospecting of natural compounds from brazilian cerrado biome plants in human cervical cancer cell lines. Int J Mol Sci. 10.3390/ijms22073383 33806119 10.3390/ijms22073383 PMC8036847

[CR60] Rossetto I, Santos F, Kido L et al (2023) Tempol differential effect on prostate cancer inflammation: in vitro and in vivo evaluation. Prostate 83:403–415. 10.1002/pros.24473 36546327 10.1002/pros.24473

[CR61] Ru P, Steele R, Nerurkar PV et al (2011) Bitter melon extract impairs prostate cancer cell-cycle progression and delays prostatic intraepithelial neoplasia in TRAMP model. Cancer Prev Res 4:2122–2130. 10.1158/1940-6207.CAPR-11-0376 10.1158/1940-6207.CAPR-11-0376 PMC3232292 21911444

[CR62] Sailer V, von Amsberg G, Duensing S et al (2023) Experimental in vitro, ex vivo and in vivo models in prostate cancer research. Nat Rev Urol 20:158–178. 10.1038/s41585-022-00677-z 36451039 10.1038/s41585-022-00677-z

[CR63] Santos FR, Rossetto IMU, Montico F et al (2024) Differential tempol effects in prostatic cancer: angiogenesis and short- and long-term treatments. J Mol Histol 55:253–264. 10.1007/s10735-024-10187-4 38551737 10.1007/s10735-024-10187-4

[CR64] Saranyutanon S, Deshmukh SK, Dasgupta S et al (2020) Cellular and molecular progression of prostate cancer: models for basic and preclinical research. Cancers (Basel) 12:2651. 10.3390/cancers12092651 32957478 10.3390/cancers12092651 PMC7563251

[CR65] Schatten H (2018) Brief overview of prostate cancer statistics, grading, diagnosis and treatment strategies. In: Advances in Experimental Medicine and Biology. 1–14 10.1007/978-3-319-95693-0_1 30229546

[CR66] Silva VAO, Alves ALV, Rosa MN, Alves ALV, Silva LRV et al (2018) Hexane partition from Annona crassiflora Mart. promotes cytotoxity and apoptosis on human cervical cancer cell lines. Invest New Drugs 37:602–615. 10.1007/s10637-018-0657-y 30155717 10.1007/s10637-018-0657-y

[CR67] Singh CK, Chhabra G, Ndiaye MA et al (2020) Quercetin–resveratrol combination for prostate cancer management in TRAMP mice. Cancers (Basel) 12:2141. 10.3390/cancers12082141 32748838 10.3390/cancers12082141 PMC7465013

[CR68] Tummala R, Lou W, Gao AC, Nadiminty N (2017) Quercetin targets hnRNPA1 to overcome enzalutamide resistance in prostate cancer cells. Mol Cancer Ther 16:2770–2779. 10.1158/1535-7163.MCT-17-0030 28729398 10.1158/1535-7163.MCT-17-0030 PMC5716891

[CR69] Tummers B, Green DR (2017) Caspase‐8: regulating life and death. Immunol Rev 277:76–89. 10.1111/imr.12541 28462525 10.1111/imr.12541 PMC5417704

[CR70] Tuxhorn JA, Ayala GE, Smith MJ et al (2002) Reactive stroma in human prostate cancer: induction of myofibroblast phenotype and extracellular matrix remodeling. Clin Cancer Res 8:2912–2923 12231536

[CR71] Tzifi F, Economopoulou C, Gourgiotis D et al (2011) The role of BCL2 family of apoptosis regulator proteins in acute and chronic leukemias. Adv Hematol 2012:1–15. 10.1155/2012/524308 10.1155/2012/524308 PMC3173728 21941553

[CR72] Valentini E, D’Aguanno S, Di Martile M et al (2022) Targeting the anti-apoptotic Bcl-2 family proteins: machine learning virtual screening and biological evaluation of new small molecules. Theranostics 12:2427–2444. 10.7150/thno.64233 35265218 10.7150/thno.64233 PMC8899577

[CR73] Wagle NS, Nogueira L, Devasia TP et al (2025) Cancer treatment and survivorship statistics, 2025. CA Cancer J Clin 72:409–436. 10.3322/caac.70011 10.3322/caac.70011 PMC12223361 40445120

[CR74] Wang WH, Tyan YC, Chen ZS et al (2014) Evaluation of the antioxidant activity and antiproliferative effect of the jaboticaba (Myrciaria cauliflora) seed extracts in oral carcinoma cells. Biomed Res Int 2014:1–8. 10.1155/2014/185946 10.1155/2014/185946 PMC4150497 25197631

[CR75] Wu G, Tu Z, Yang F et al (2021) Evaluating the inhibitory priority of Bcl-xL to Bad, tBid and Bax by using live-cell imaging assay. Cytom Part A 99:1091–1101. 10.1002/cyto.a.24351 10.1002/cyto.a.24351 33843148

[CR76] Wyżewski Z, Gregorczyk-Zboroch KP, Mielcarska MB et al (2025) Bid protein: a participant in the apoptotic network with roles in viral infections. Int J Mol Sci 26:2385. 10.3390/ijms26062385 40141030 10.3390/ijms26062385 PMC11942203

[CR77] Yaacoub K, Pedeux R, Tarte K, Guillaudeux T (2016) Role of the tumor microenvironment in regulating apoptosis and cancer progression. Cancer Lett 378:150–159. 10.1016/j.canlet.2016.05.012 27224890 10.1016/j.canlet.2016.05.012

[CR78] Yang C, Gundala SR, Mukkavilli R et al (2015) Synergistic interactions among flavonoids and acetogenins in Graviola (Annona muricata) leaves confer protection against prostate cancer. Carcinogenesis 36:656–665. 10.1093/carcin/bgv046 25863125 10.1093/carcin/bgv046 PMC4566098

[CR79] Yuan J, Ofengeim D (2024) A guide to cell death pathways. Nat Rev Mol Cell Biol 25:379–395. 10.1038/s41580-023-00689-6 38110635 10.1038/s41580-023-00689-6

[CR80] Zar JH (1999) Biostatistical analysis, 4th Editio. Upper Saddle Rive

[CR81] Zhou Y, Bolton EC, Jones JO (2015) Androgens and androgen receptor signaling in prostate tumorigenesis. J Mol Endocrinol 54:R15–R29. 10.1530/JME-14-0203 25351819 10.1530/JME-14-0203

[CR82] Zorofchian Moghadamtousi S, Karimian H, Rouhollahi E et al (2014) Annona muricata leaves induce G1 cell cycle arrest and apoptosis through mitochondria-mediated pathway in human HCT-116 and HT-29 colon cancer cells. J Ethnopharmacol 156:277–289. 10.1016/j.jep.2014.08.011 25195082 10.1016/j.jep.2014.08.011

