# Supplementary material for: Araticum (Annona crassiflora) seed extract as a chemopreventive agent against prostate cancer: activation of extrinsic and intrinsic apoptotic pathways in a preclinical model
Source: J Mol Histol. 2026 Jun 13;57(4):195. doi: 10.1007/s10735-026-10859-3 (PMC13264554; doi:10.1007/s10735-026-10859-3)

Supplementary material

1. **General Toxicity Analysis**

1.1 Body Weight Evaluation

The animals' body weight was measured using a digital scale at the beginning of the experiment and subsequently at weekly intervals throughout the experimental period, and it was used as an weight growth indicator, overall health status, and treatment tolerability among the experimental groups (Ukwubile et al., 2025).

1.2 Liver Histopathological Analysis

Histopathological evaluation of the liver was performed based on the protocol described by modified Lamas et al. (2018). Hepatic samples from three animals per experimental group were collected and fixed in Bouin's solution for 24 h. Subsequently, the tissues were washed in 70% ethanol until complete removal of the fixative, followed by dehydration in an ascending alcohol series, clearing in xylene, and embedding in plastic polymer (Paraplast Plus, St. Louis, USA). The embedded samples were sectioned into 5 µm–thick slices using a Hyrax M60 microtome (Zeiss, Munich, Germany) and stained with hematoxylin and eosin for morphological evaluation. Analyses were performed using images obtained with a Nikon Eclipse E-400 optical microscope (Nikon, Tokyo, Japan), using the NIS-Elements/Image and Image Pro-Plus software. For quantification, a grid with 171 intersection points was applied to 10 randomly selected images per animal at 400× magnification. The histopathological evaluation focused on identifying morphological alterations associated with hepatic toxicity, including the number of mononucleated and binucleated hepatocytes, total hepatocyte cytoplasmic area, presence of lipid content, inflammatory infiltrate, and hepatocellular ballooning. Elements that did not fit these characteristics were not counted (Lamas et al., 2018).

RESULTS

Daily administration of ASE (100 mg/kg) or its dilution vehicle (water containing 10% DMSO) did not promote significant changes in the body weight of TRAMP mice. Over the four-week experimental period, a progressive and similar weight gain was observed among the evaluated groups (Figure S1 A and B).

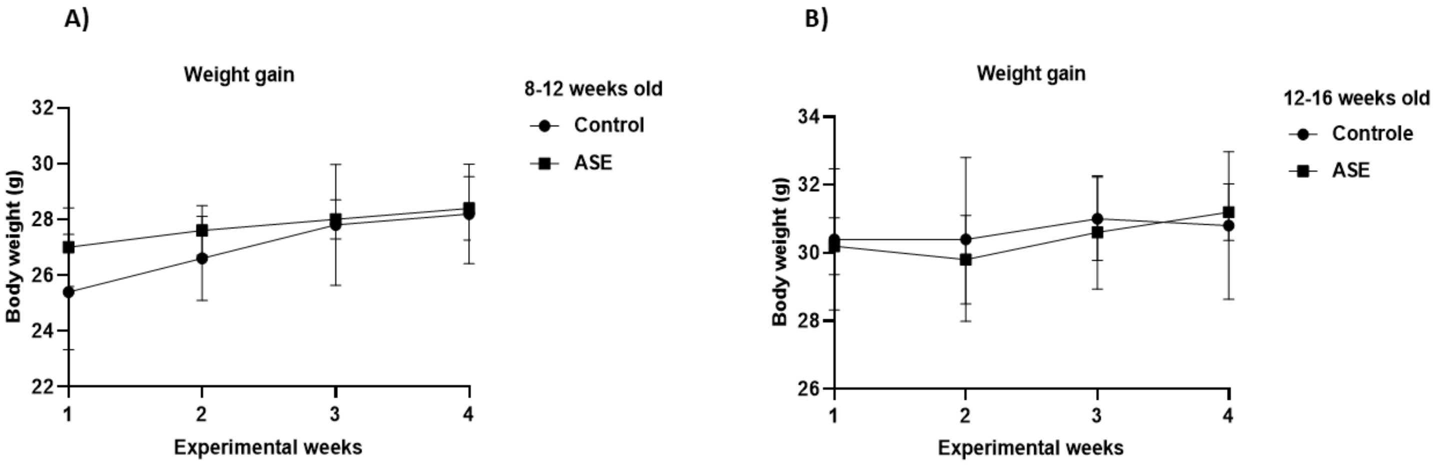

Figure S1. Body weight gain curves of the control and ASE groups over the four-week experimental period. (A) 8–12-week-old group. (B) 12–16-week-old group. Data are presented as mean ± standard deviation.

Histopathological analysis of the liver demonstrated that administration of ASE, or of the extract dilution vehicle (water containing 10% DMSO), for four weeks did not compromise liver morphological integrity, since no significant histopathological alterations were observed in either experimental group. In the 8–12-week-old group, no differences were observed in the frequency of mononucleated or binucleated hepatocytes between the control and ASE-treated groups. The cytoplasmic integrity of hepatocytes remained preserved in both groups, with no evidence of vacuolization or relevant degenerative alterations (Figure S2).

Isolated findings of hepatic lipid content were identified in individual animals from both groups (control: 0.05%; ASE: 0.05%). Similarly, mild inflammatory infiltrate was observed in both the control and ASE treated groups (0.04% and 0.05%, respectively), at low magnitude and without differences between groups. Additionally, no signs of cellular ballooning were observed in the evaluated groups.

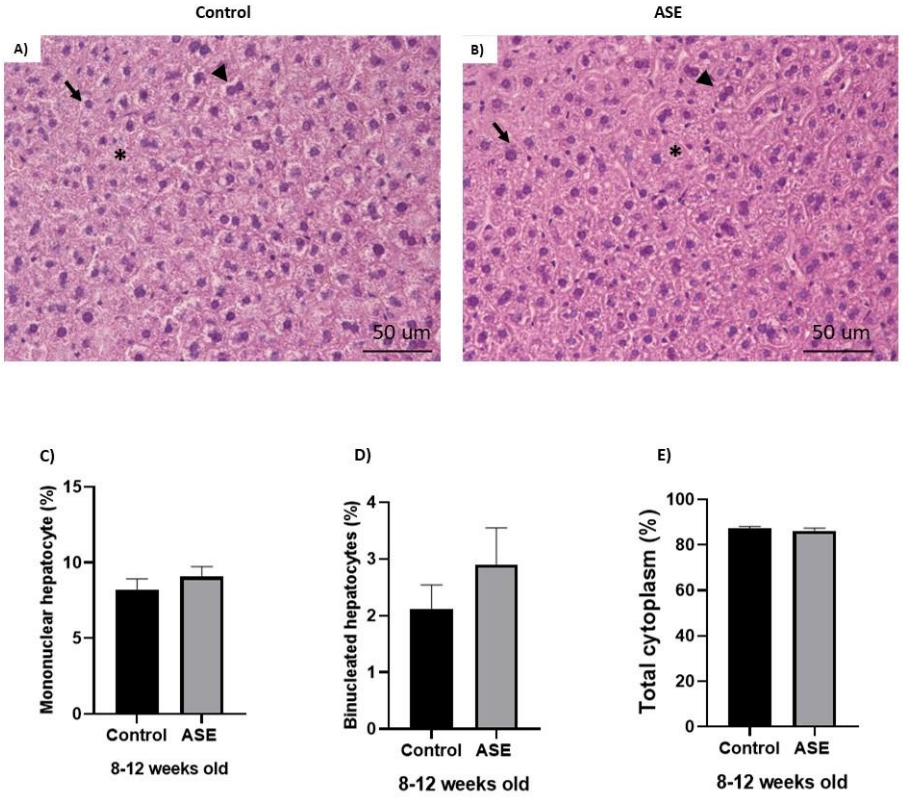

Figure S2. (A–B) Photomicrographs of liver tissue stained with hematoxylin and eosin. (A) Control group, 8–12 weeks of age. (B) ASE group, 8–12 weeks of age. Arrows indicate mononucleated hepatocytes, arrowheads indicate binucleated hepatocytes, and the asterisk indicates the cytoplasmic area. (C–E) Quantitative histopathological analysis: (C) mononucleated hepatocytes (%), (D) binucleated hepatocytes (%), and (E) total cytoplasm (%). Data are presented as mean ± standard deviation.

Similar results were observed for the 12–16-week-old group; no significant differences were found between the control and ASE groups in the proportion of mononucleated hepatocytes, binucleated hepatocytes, or total cytoplasm (Figure S3). Discrete hepatic lipid content and inflammatory infiltrate were observed in both groups (control: 0.01%; ASE: 0.01%), with no evidence of hepatocellular ballooning.

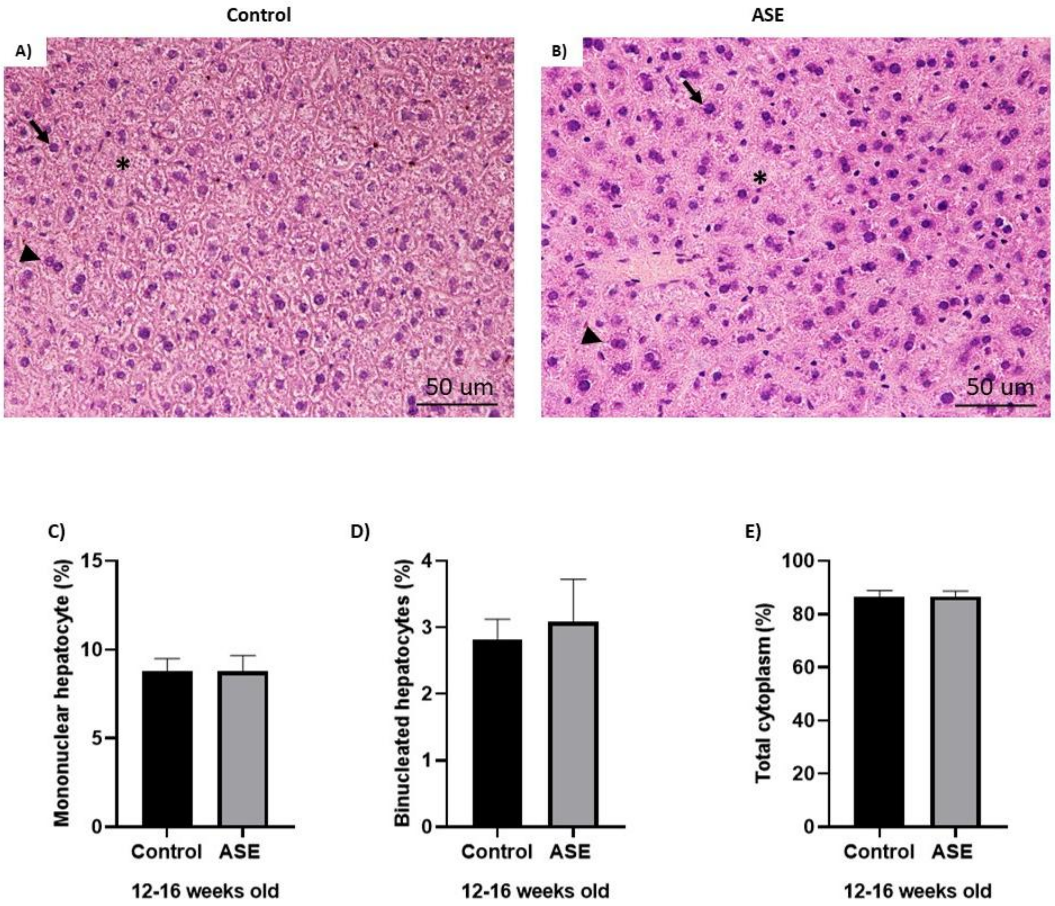

Figure S3. (A–B) Photomicrographs of liver tissue stained with hematoxylin and eosin (H&E). (A) Control group, 12–16 weeks of age. (B) ASE group, 12–16 weeks of age. Arrows indicate mononucleated hepatocytes, arrowheads indicate binucleated hepatocytes, and the asterisk indicates the cytoplasmic area. (C–E) Quantitative histopathological analysis: (C) mononucleated hepatocytes (%), (D) binucleated hepatocytes (%), and (E) total cytoplasm (%). Data are presented as mean ± standard deviation.

2. **Immunohistochemical detection of Bid, Caspase-3, Bcl-xL, PCNA, and AR in the dorsolateral lobe of TRAMP mice and their respective negative controls.**

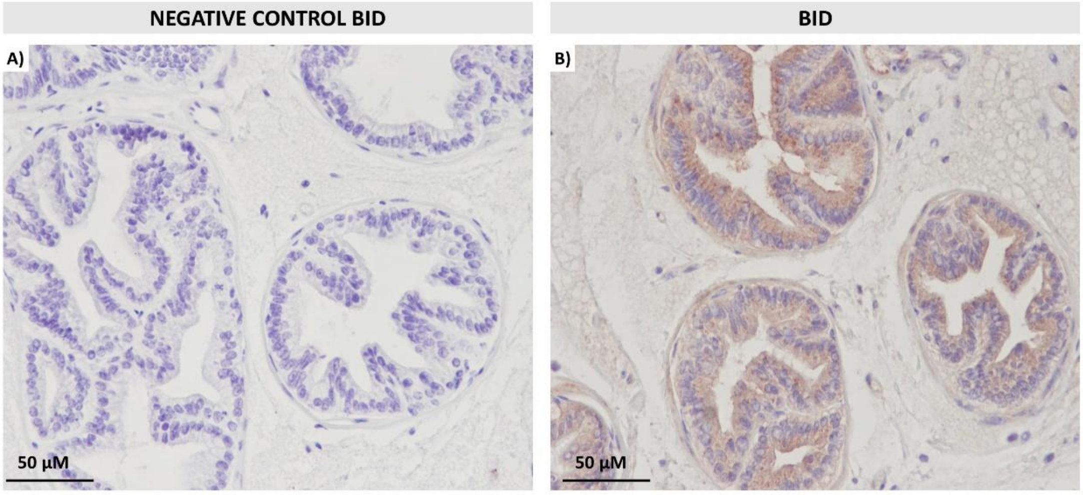

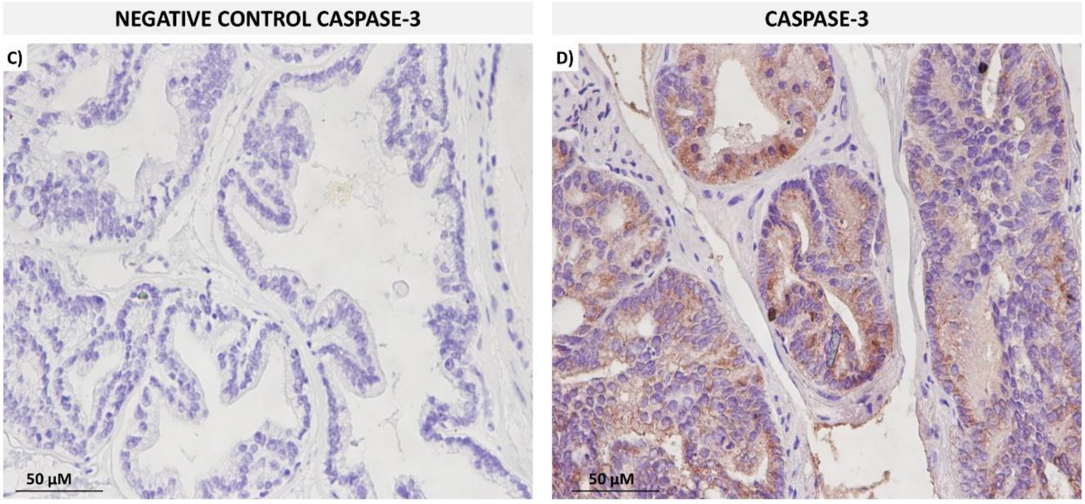

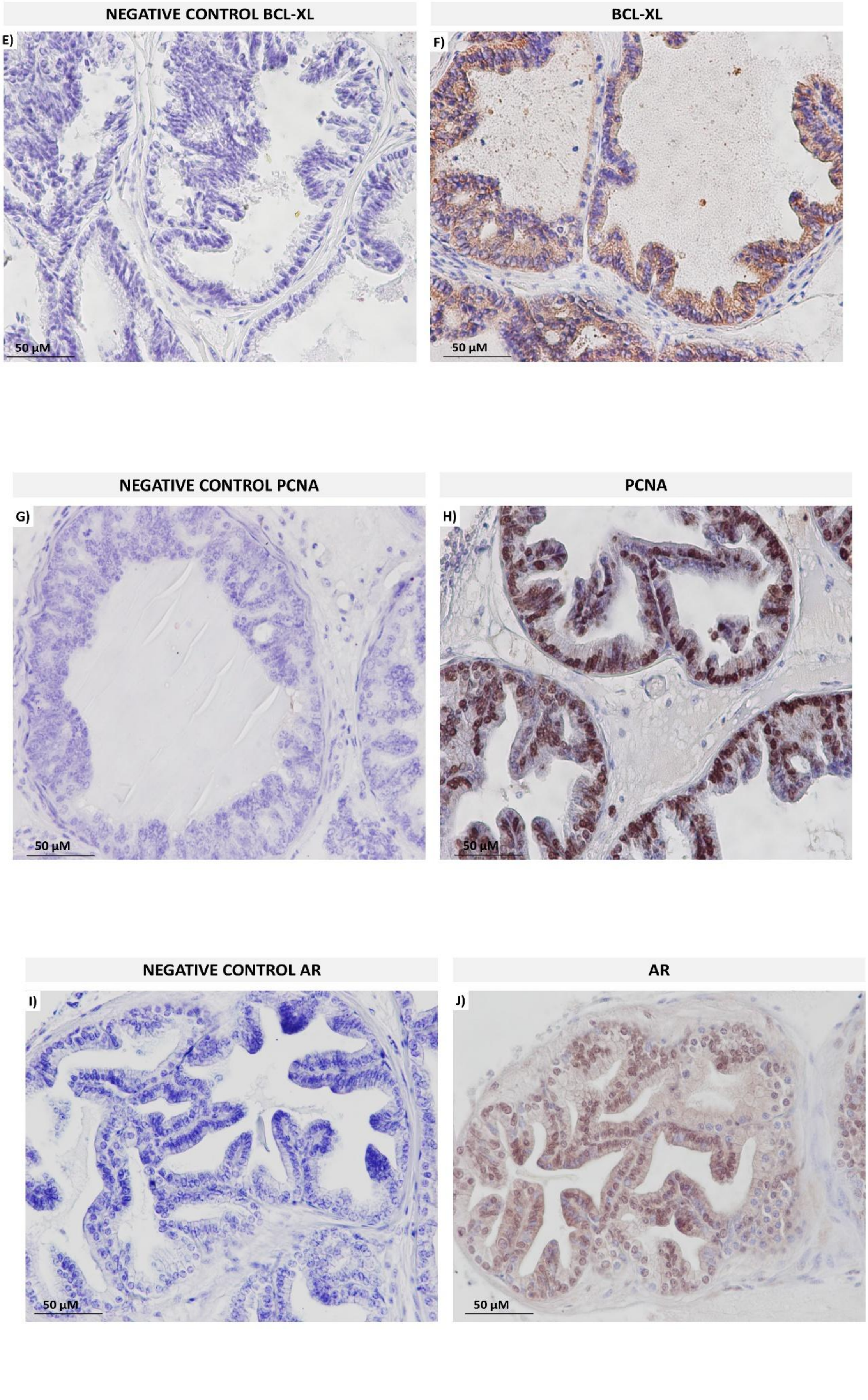
NEGATIVE CONTROL BCL-XL
BCL-XL
E)
F)
50 µM
50 µM
NEGATIVE CONTROL PCNA
PCNA
G)
H)
50 µM
50 µM
NEGATIVE CONTROL AR
AR
I)
J)
50 µM
50 µM

Uncropped Western Blot Membranes

Full-length, uncropped Western blot membranes corresponding to all proteins presented in the main figures are provided in this supplementary document, as requested by the reviewers. For each target protein, the complete membrane image is shown together with the respective β-actin loading control and the corresponding Ponceau S staining used to verify equal protein loading and transfer efficiency. The lanes are presented in the same order as displayed in the main figures, and no additional image processing was performed other than uniform brightness and contrast adjustments applied to the entire image.

BAX. Membrane 9. 21.11.24

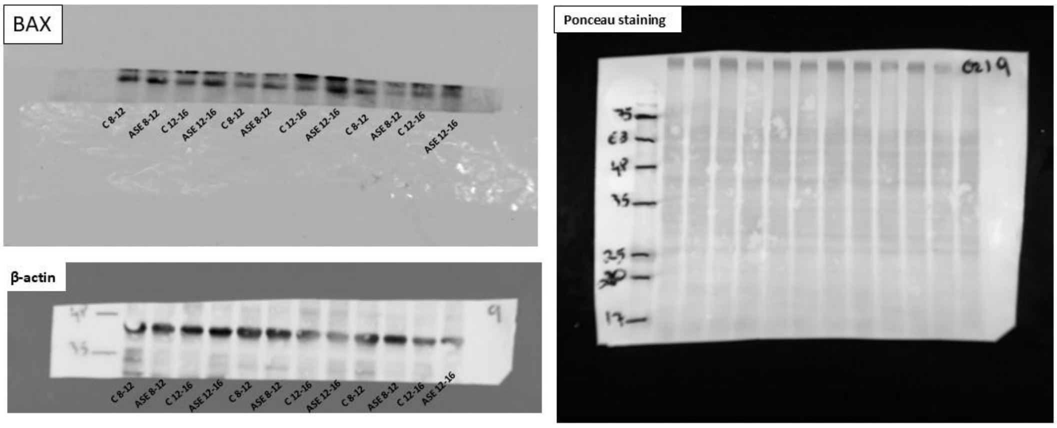

BAD. Membrane 10. 19.11.24

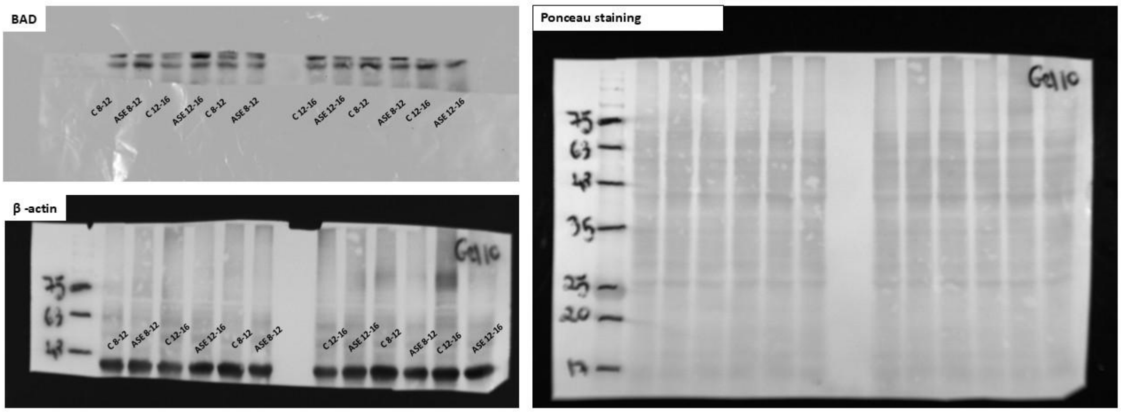

BID. Membrane 33. 23.01.25

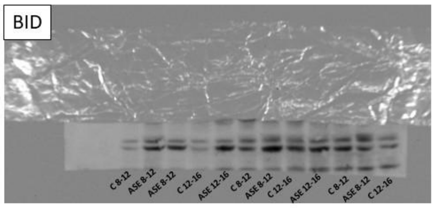

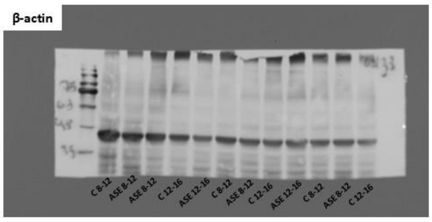

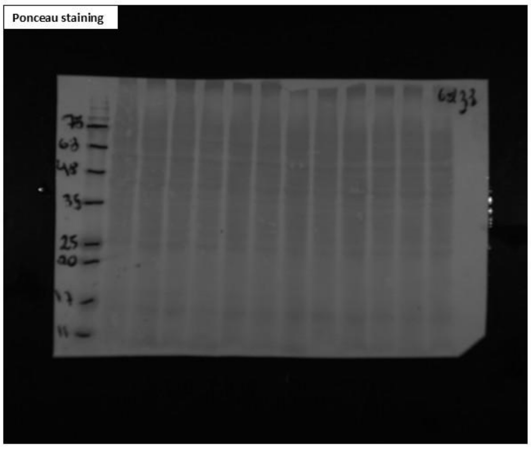

Procaspase-9. Membrane 16. 28.11.24

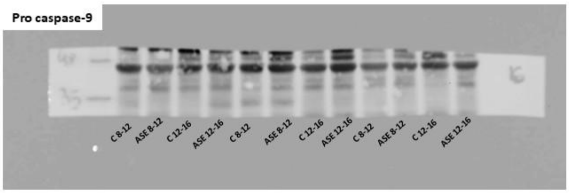

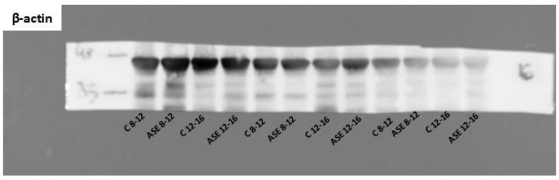

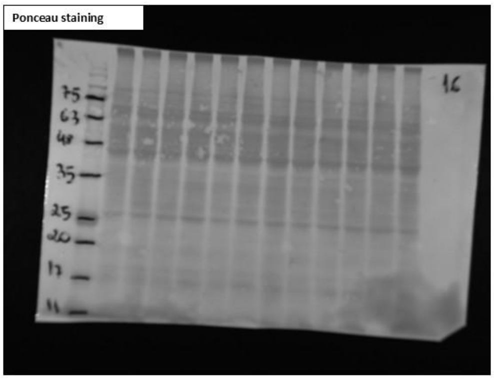

Caspase-9. Membrane 34. 22.01.25

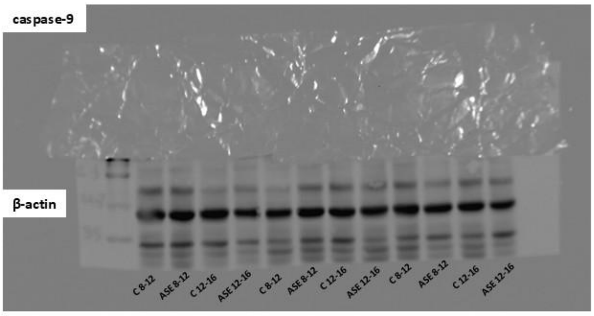

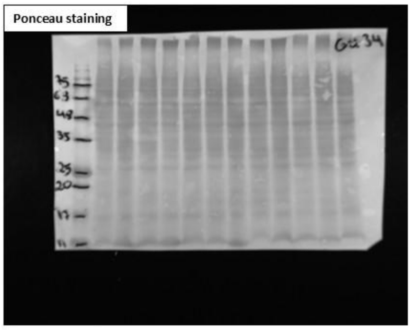

Caspase-8. Membrane 10. 26.11.24

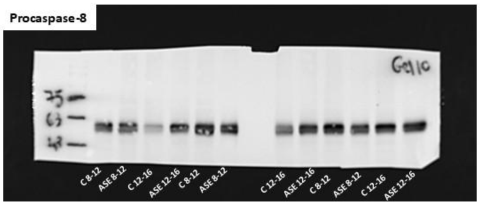

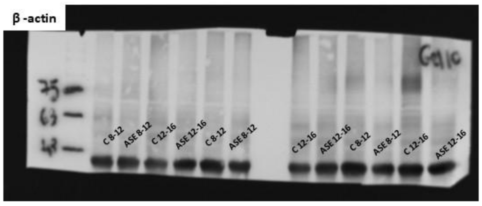

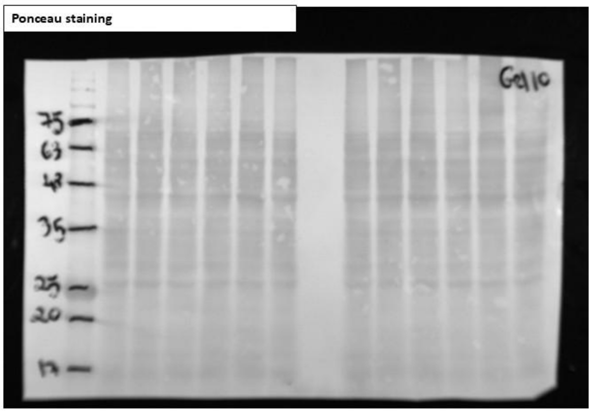

Pro-caspase-3. Membrane 34. 31.01.25

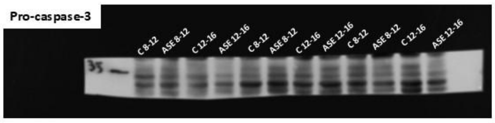

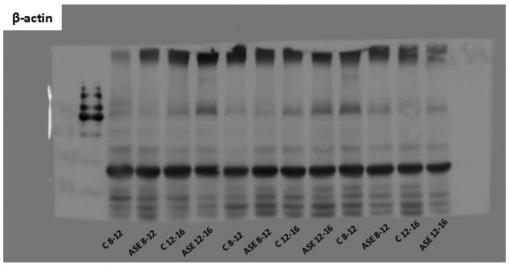

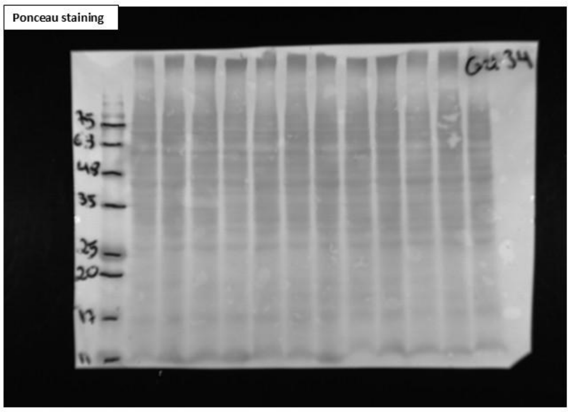

Caspase-3. Membrane 17. 28.11.24

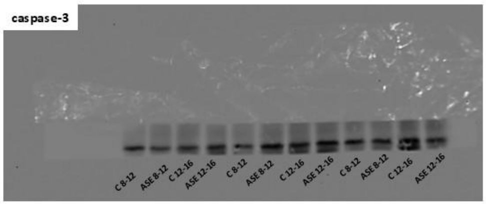

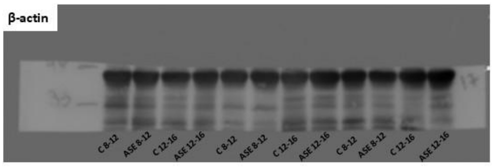

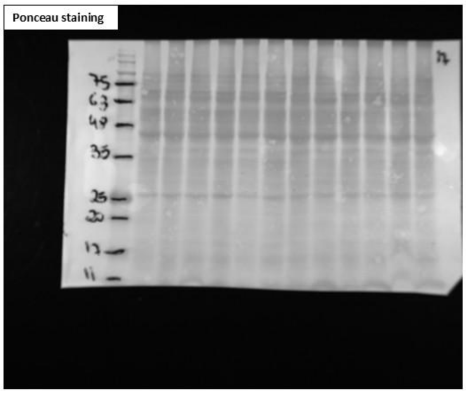

BCL-2. Membrane 34. 28.01.25

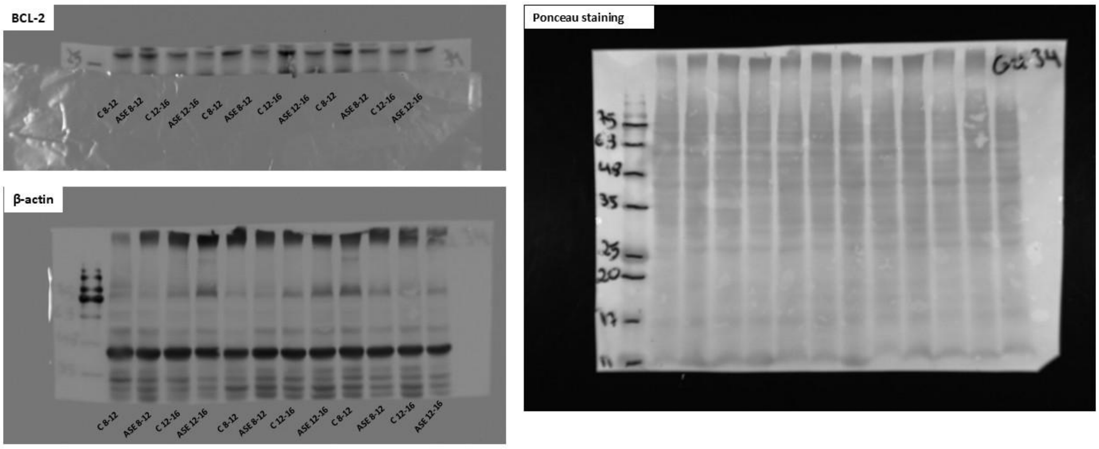

BCL-XL. Membrane 27. 17.01.25

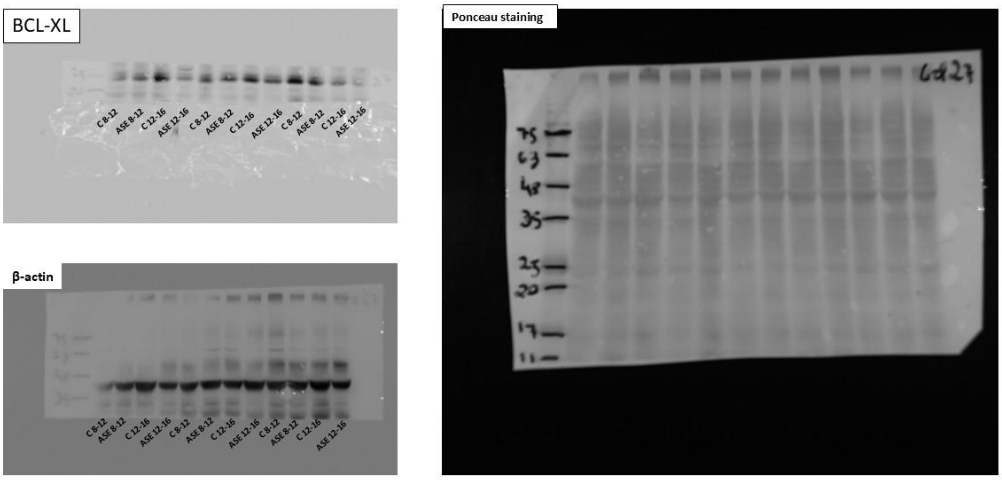

MCL-1. Membrane 30. 16.01.25

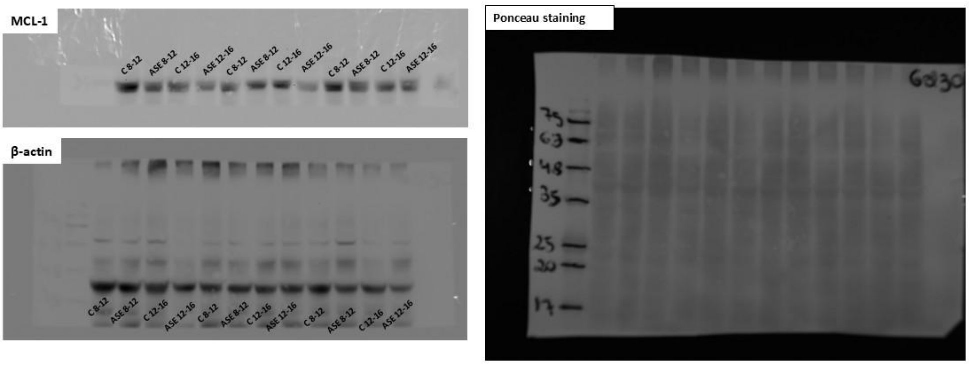

PCNA. Membrane 29. 17.01.25

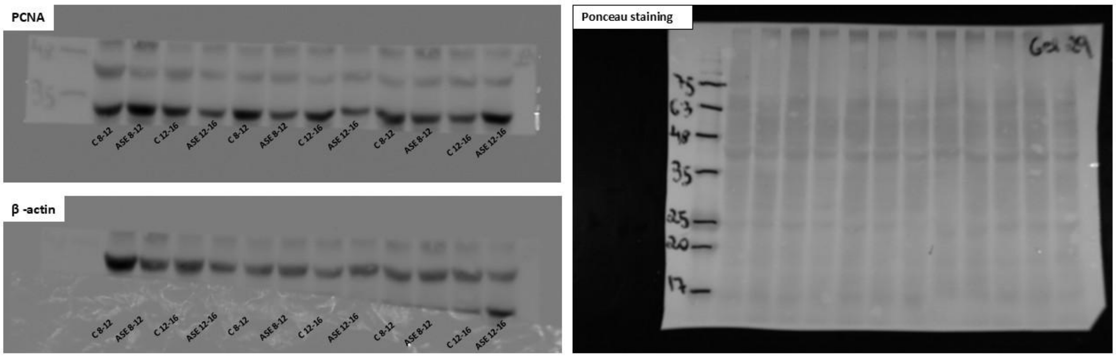

AR. Membrane 24. 04.12.24

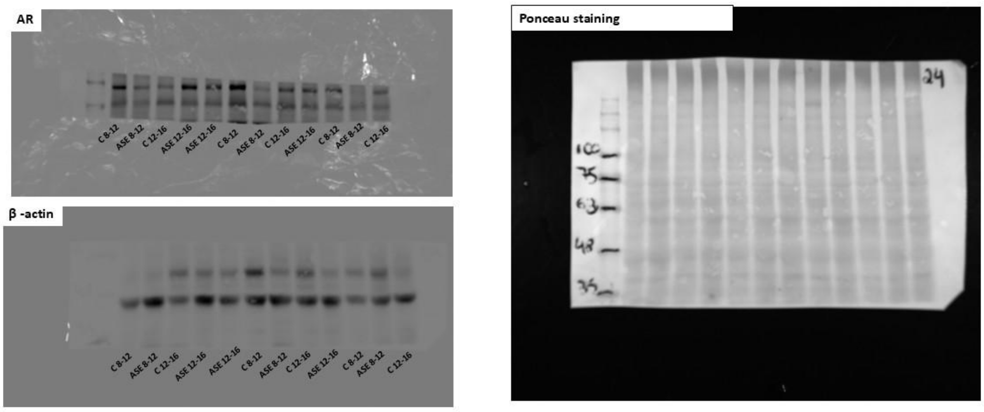

Supplement: Supplementary file 1 — Supplementary Material 1 [file 10735_2026_10859_MOESM1_ESM.pdf]
